# Supplementary material for: Zoledronate in the prevention of Paget’s (ZiPP): protocol for a randomised trial of genetic testing and targeted zoledronic acid therapy to prevent SQSTM1-mediated Paget’s disease of bone
Source: BMJ Open. 2019 Sep 4;9(9):e030689. doi: 10.1136/bmjopen-2019-030689 (PMC6731944; doi:10.1136/bmjopen-2019-030689)
Supplement: Author's manuscript [file bmjopen-2019-030689.draft_revisions.pdf]

BMJ Open is committed to open peer review. As part of this commitment we make the peer review history of every article we publish publicly available.

When an article is published we post the peer reviewers' comments and the authors' responses online. We also post the versions of the paper that were used during peer review. These are the versions that the peer review comments apply to.

The versions of the paper that follow are the versions that were submitted during the peer review process. They are not the versions of record or the final published versions. They should not be cited or distributed as the published version of this manuscript.

BMJ Open is an open access journal and the full, final, typeset and author-corrected version of record of the manuscript is available on our site with no access controls, subscription charges or pay-per-view fees (http://bmjopen.bmj.com).

If you have any questions on BMJ Open's open peer review process please email info.bmjopen@bmj.com

BMJ Open

Zoledronate in the prevention of Paget's (ZiPP): Protocol for a randomised trial of genetic testing and targeted Zoledronic acid therapy to prevent SQSTM1-mediated Paget's disease of bone.

Journal:	*BMJ Open*
Manuscript ID	bmjopen-2019-030689
Article Type:	Protocol
Date Submitted by the Author:	29-Mar-2019

Complete List of Authors:	Cronin, Owen; University College Cork National University of Ireland, Department of Medicine; University College Cork APC Microbiome Institute,<br>Forsyth, Laura; University of Edinburgh College of Medicine and Veterinary Medicine<br>Lewis, Steff; University of Edinburgh, Public Health Sciences<br>Keerie, Catriona; University of Edinburgh College of Medicine and Veterinary Medicine<br>Walker, Allan; University of Edinburgh College of Medicine and Veterinary Medicine<br>Porteous, Mary; University of Edinburgh College of Medicine and Veterinary Medicine<br>Cetnarskyj, Roseanne; University of Dundee - Fife Campus<br>Goodman, Kirsteen; Glasgow Caledonian University School of Nursing Midwifery and Community Health<br>Ranganath, Lakshminarayan; University of Liverpool<br>Selby, Peter; Manchester Royal Infirmary<br>Hampson, Geeta; King's College London School of Medical Education<br>Chandra, Rama; King's College Hospital<br>Ho, Shu; Robert Jones and Agnes Hunt Orthopaedic and District Hospital NHS Trust<br>Tobias, Jon ; University of Bristol, Musculoskeletal Research Unit, School of Clinical Sciences<br>Young-Min, Steven; Portsmouth Hospitals NHS Trust<br>McKenna, Malachi; University College Dublin<br>Crowley, Rachel; University College Dublin<br>Fraser, William ; Department of Medicine Norwich Medical School, Faculty of Medicine and Health Sciences, University of East Anglia, Norwich Research Park,<br>Gennari, Luigi; University of Siena Faculty of Medicine and Surgery<br>Nuti, Ranuccio; University of Siena Faculty of Medicine and Surgery<br>Brandi, Maria Luisa; University Hospital Careggi<br>Del Pino-Montes, Javier; University Hospital of Salamanca<br>Devogelaer, Jean-Pierre; Cliniques universitaires Saint-Luc<br>DURNEZ, Anne; Cliniques Universitaires Saint-Luc, Rheumatology; Algemeen Ziekenhuis Jan Portaels<br>Isaia, Giancarlo; Università degli Studi di Torino<br>Di Stefano, Marco; Università degli Studi di Torino

	Guanabens, Nuria; University of Barcelona Faculty of Medicine<br>Blanch, Josep; Hospital del Mar<br>Seibel, Markus; University of Sydney, Concord Hospital<br>Walsh, John; Sir Charles Gairdner Hospital, Department of Endocrinology and Diabetes<br>Kotowicz, Mark; Barwon Health, Department of Endocrinology and Diabetes<br>Nicholson, Geoffrey; University of Queensland, Rural Clinical School<br>Duncan, Emma; University of Queensland<br>Major, Gabor ; Royal Newcastle Centre<br>Horne, Anne; University of Auckland, Department of Medicine<br>Gilchrist, Nigel; Princess Margaret Hospital<br>Boers, Maarten; VU University Medical Center, Clinical Epidemiology and Biostatistics<br>Murray, Gordon; University of Edinburgh, Public Health Sciences<br>Wilkinson, Diana; The Paget's Association<br>Russell, Graham; University of Oxford<br>Charnock, Keith; The Paget's Association<br>Ralston, Stuart; University of Edinburgh,

Keywords:	Calcium & bone < DIABETES & ENDOCRINOLOGY, GENETICS, RHEUMATOLOGY

SCHOLARONE™
Manuscripts

Zoledronate in the prevention of Paget's (ZiPP): Protocol for a randomised trial of genetic testing and targeted Zoledronic acid therapy to prevent *SQSTM1*-mediated Paget's disease of bone.

Owen Cronin[1], Laura Forsyth[2], Kirsteen Goodman[2 §] Steff Lewis[2], Catriona Keerie[2], Allan Walker[2], Mary Porteous[3], Roseanne Cetnarskyj[3,4], Lakshminarayan R Ranganath[5], Peter L Selby[6], Geeta Hampson[7], Rama Chandra[8], Shu Ho[89], Jon H Tobias[10], Steven Young-Min[11], Malachi McKenna[12,13], Rachel Crowley[12,13], William D. Fraser[14], Luigi Gennari[15], Ranuccio Nuti[15], Maria Luisa Brandi[16], Javier del Pino-Montes[17], Jean-Pierre Devogelaer[18], Anne Durnez[18], Giancarlo Isaia[19], Marco Di Stefano[19], Nuria Guanabens[20], Josep Blanch[21], Markus J Seibel[22], John Walsh[23], Mark A Kotowicz[24], Geoffrey C Nicholson[25], Emma Duncan[26], Gabor Major[27], Anne Horne[28], Nigel Gilchrist[29], Maarten Boers[30], Gordon D Murray[31], Keith Charnock[32], Diana Wilkinson[32], R Graham G Russell[33], Stuart H. Ralston[1,2,34]

1. Rheumatic Diseases Unit, Western General Hospital, Crewe Road South, Edinburgh, EH4 2XU, United Kingdom.
2. Edinburgh Clinical Trials Unit, Usher Institute, University of Edinburgh, Nine Edinburgh Bioquarter, Little France Road, Edinburgh, EH16 4UX, United Kingdom.
3. South East Scotland Molecular Genetics Service, Western General Hospital; Edinburgh EH4 2XY, United Kingdom.
4. School of Nursing & Health Sciences, University of Dundee, Kirkcaldy Campus. Kirkcaldy, KY2 5YS, UK.
5. Department of Clinical Biochemistry and Metabolic Medicine, University of Liverpool, Daulby Street, L69 3GA, United Kingdom.
6. Manchester Royal Infirmary, Oxford Road, Manchester, M13 9WL, United Kingdom.
7. Department of Chemical Pathology and Metabolic Bone Clinic, Department of Rheumatology, St. Thomas' Hospital, Westminster Bridge Road, London, SE1 7EH, United Kingdom.
8. Clinical Biochemistry, King's College Hospital, Denmark Hill, London, SE5 9RS .
9. The Robert Jones and Agnes Hunt Orthopaedic and District Hospital, Twmpath Lane, Oswestry, SY10 7AG, United Kingdom.
10. Musculoskeletal Research Unit, Translational Health Sciences, Bristol Medical School, University of Bristol, Bristol, BS10 5NB, United Kingdom.
11. Department of Rheumatology, Queen Alexandra Hospital, Southwick Hill Road, Cosham, PO6 3LY, United Kingdom.
12. Endocrinology Department, St. Vincent's University Hospital, Elm Park, Dublin 4, Republic of Ireland.
13. University College Dublin, Belfield, Dublin 4, D04 V1W8, Republic of Ireland.
14. Norwich Medical School, Faculty of Medicine and Health Sciences, University of East Anglia, Norwich, NR4 7TJ, United Kingdom.
15. Department of Medicine, Surgery and Neurosciences, University of Sienna, Policlinico Le Scotte, Viale Bracci, Sienna, 53100, Italy.
16. University Hospital of Careggi, Department of Internal Medicine, Viale Peircaccine 6, Florence 50139, Italy.
17. University Hospital of Salamanca, Rheumatology Department, San Vincente 54-132, Salamanca, 370007, Spain.

For peer review only

For peer review only - http://bmjopen.bmj.com/site/about/guidelines.xhtml

[18] Clinique Universitaires Saint-Luc, n-10 Avenue Hippocrate, Brussels, B-1200, Belgium.
[19] Geriatrics and Metabolic Bone Diseases, AOU San Giovanni Battista di Torino Corso, Bramante 88, Torino, 10126, Italy.
[20] Department of Rheumatology, Hospital Clinic, CIBERehd, C/Villarroel 170, University of Barcelona, Barcelona, 8036, Spain.
[21] Hospital del Mar, Passeig Maritim, 08003 Barcelona, Spain.
[22] Bone Research Program, ANZAC Research Institute, and Department of Endocrinology and Metabolism, Concord Repatriation General Hospital, Concord, Sydney, NSW 2139, Australia.
[23] Department of Endocrinology and Diabetes, Sir Charles Gairdner Hospital, Nedlands, WA 6009, Australia and the Medical School, The University of Western Australia, Crawley, Western Australia 6009.
[24] Deakin University, Geelong, Victoria, Australia; Barwon Health, Geelong, Victoria, Australia; and the Department of Medicine-Western Health, Melbourne Medical School, The University of Melbourne, Victoria, Australia.
[25] Rural Clinical School Research Centre, Darlin Heights, the University of Queensland, Queensland 4350, Australia.
[26] Endocrinology Department, Royal Brisbane and Woman's Hospital, Butterfield Street, Herston QLD 4029, Australia.
[27] Rheumatology, Bone and Joint Institute, Royal Newcastle Center, 2 Lookout Road, New Lambton Heights, NSW 2305, Australia.
[28] Department of Medicine, Faculty of Medical and Health Sciences, University of Auckland, Auckland 1142, New Zealand.
[29] The CGM Research Trust, The Princess Margaret Hospital, PO Box 731, Christchurch, New Zealand.
[30] Amsterdam Rheumatology and Immunology Center, ARC, VU University Medical Center, Amsterdam, the Netherlands.
[31] Usher Institute of Population Health Sciences and Informatics, University of Edinburgh, Edinburgh, UK.
[32] The Paget's Association, Moorside Road, Manchester, M27 0EW, UK
[33] Botnar Research Centre, Nuffield Department of Orthopaedics, Rheumatology & Musculoskeletal Sciences, University of Oxford, Oxford, UK and the Mellanby Centre for Bone Research, University of Sheffield, Sheffield, UK
[34] Centre for Genomic and Experimental Medicine, Institute of Genetics and Molecular Medicine, University of Edinburgh, Western General Hospital, Edinburgh, EH 2XU, United Kingdom.

Corresponding author:
Professor Stuart H Ralston
Centre for Genomic and Experimental Medicine,
Institute of Genetics and Molecular Medicine,
University of Edinburgh,
Western General Hospital,
Edinburgh, EH 2XU, United Kingdom.
Email: stuart.ralston@ed.ac.uk

[§] Present address:
NMAHP Research Unit
Glasgow Caledonian University
Cowcaddens Road, Glasgow G4 0BA
UK

For peer review only

ABSTRACT

Introduction: Paget's disease of bone (PDB) is characterised by increased and disorganised bone remodelling affecting one or more skeletal sites. Complications include bone pain, deformity, deafness and pathological fractures. Mutations in *SQSTM1* are strongly associated with development of PDB. Bisphosphonate therapy can improve bone pain in PDB, but there is no evidence that treatment alters the natural history of PDB or prevents complications. The ZiPP trial will determine if prophylactic therapy with the bisphosphonate zoledronic acid (ZA) can delay or prevent the development of PDB in people who carry *SQSTM1* mutations.

Methods and analysis: People with a family history of PDB aged >30 years who test positive for *SQSTM1* mutations are eligible to take part. At the baseline visit participants are screened for the presence of lesions by radionuclide bone scan. Biochemical markers of bone turnover will be measured and questionnaires completed to assess pain, health related quality of life (HRQoL), anxiety and depression. Participants will be randomised to receive a single intravenous infusion of 5mg ZA or placebo and followed up annually for between 4 and 8 years at which point baseline assessments will be repeated. The primary endpoint will be new bone lesions assessed by radionuclide bone scan. Secondary endpoints will include changes in biochemical markers of bone turnover, pain, HRQoL, anxiety, depression and PDB-related skeletal events.

Ethics and Dissemination: The study was approved by the Fife and Forth Valley Research Ethics Committee on 22nd December 2008 (08/S0501/84). Following completion of the trial, a manuscript will be submitted to a peer-reviewed journal. The results of this trial will inform clinical practice by determining if early intervention with ZA in pre-symptomatic individuals with *SQSTM1* mutations can prevent or slow the development of bone lesions with an adverse event profile that is acceptable.

Trial registration number: ISRCTN11616770; Pre-results.

Abstract: 298 words

Total word count: 3567

For peer review only

Strengths and limitations

- This randomised placebo controlled trial will test the hypothesis that genetic testing coupled with targeted intervention with the bisphosphonate zoledronic acid can modify the development and progression of bone lesions secondary to Paget's disease .
- While participants have a high genetic risk of developing Paget's disease due to *SQSTM1* mutations, the proportion of untreated individuals who will develop Paget's during follow up remains uncertain
- Longer term follow up will be required to evaluate the effect of the intervention on complications of Paget's disease such as bone pain, deformity and pathological fractures.

Keywords:
Paget's Disease of Bone; bisphosphonate; Zoledronic acid; SQSTM1, genetics; randomized controlled trial.

For peer review only

BACKGROUND

Paget's disease of bone (PDB) is characterised by areas of increased and disorganised bone turnover affecting one or more skeletal sites. Many affected individuals develop complications such as bone pain, deformity, deafness, pathological fracture and osteoarthritis. Genetic factors play an important role in PDB and *SQSTM1* mutations are an important predisposing factor, occurring in up to 50% of patients with a family history of PDB and up to 10% of people who are unaware of having a family history (1-6). Carriers of *SQSTM1* mutations have more severe disease with an earlier age at onset than patients who do not carry mutations (7). While cross-sectional studies indicate that the penetrance of PDB in *SQSTM1* mutation carriers is about 80% above the age of 70 years (8) there has been only one prospective study of disease evolution in these individuals. In this study, which involved 10 families with *SQSTM1* mutations 4/23 (17%) of mutation carriers had developed the PDB disease by the age of 50 as assessed by radionuclide bone scanning, but the age at diagnosis was delayed compared with their parents' (9). Bisphosphonates are considered to be the treatment of choice for PDB, and within the bisphosphonates, zoledronic acid (ZA) is most likely to give a favourable response in terms of bone pain (10). Zoledronic acid has a very long duration of action in patients with established PDB with effects that can last for more that 6 years (11). The main indication for bisphosphonate treatment in PDB is bone pain thought to be due to increased metabolic activity of the disease (10, 12). Although bisphosphonates are highly effective at supressing elevated bone turnover in PDB, there is no evidence that giving bisphosphonate treatment with the primary aim of supressing elevated bone turnover is of clinical benefit (12). There is also no evidence that treatment can prevent complications of PDB such as fracture, bone deformity, deafness or secondary osteoarthritis (13, 14). The Zoledronic acid in prevention of Paget's disease (ZiPP) trial has been designed to determine if prophylactic treatment with ZA is effective at preventing the development or progression of bone lesions with the characteristic features of PDB in asymptomatic *SQSTM1* mutation carriers

Good clinical practice

The study will be carried out according to the principles of the International Conference on Harmonisation Tripartite Guideline for Good Clinical Practice and local guidance and regulations

Consolidated standards of reporting trials

The results of the trial will be reported in accordance with the Consolidated Standards Of Reporting Trials (CONSORT) statement.

Aim

To improve clinical outcome in PDB by exploring whether genetic testing for *SQSTM1* mutations coupled with prophylactic treatment with ZA, can prevent of slow the development of bone lesions,

For peer review only - http://bmjopen.bmj.com/site/about/guidelines.xhtml

prevent skeletal complications and favourably modify pain and quality of life in those genetically at risk because of a positive family history.

Objectives

The study objectives are summarised in Table 1 and described in more detail below

The primary objective is:

To quantify the number of subjects in each treatment group who develop new bone lesions with the characteristics of PDB as assessed by radionuclide bone scan. The presence and location of lesions will be assessed by imaging experts blinded to treatment allocation. A new bone lesion will be defined as evidence of involvement of a new bone or part of a previously affected bone at the end-of-study visit which was not thought to be involved at the baseline visit.

The secondary objectives are:

To quantify the number of new bone lesions and change in activity of existing bone lesions present at baseline; to evaluate the effects of treatment on skeletal events related to PDB; to evaluate the effects of treatment on biochemical markers of bone resorption and bone formation; and to evaluate the effects of treatment on health related quality of life, anxiety and depression and the presence, localisation and severity of pain.

Outcome measures

The schedule of assessments and outcome measures which will be collected during the study are summarised in Table 2 and are discussed individually in more detail below.

Bone lesions

These will be assessed by evaluation of Tc^{99} radionuclide bone scans images. Participants thought to have PDB-like bone lesions on scan may have further imaging performed by x-ray, CT scan or MRI scan if the local investigator considers it clinically indicated. Anonymised bone scan and x-ray images will be uploaded on to the study database for review. All scans will be reviewed by an imaging expert blinded to treatment allocation. A proportion of images will reviewed by a second imaging expert, also blinded to treatment allocation, to evaluate concordance between observers. The images selected will include all of those considered by the primary imaging expert to represent PDB-like lesions. In the event that the experts disagree on a specific image a third imaging expert (also blinded to treatment allocation) will be asked to adjudicate.

Clinical assessments

Participants will undergo a physical examination and at the baseline visit including blood pressure and pulse. Participants will be evaluated clinically at the end of trial visit for any symptoms or signs of skeletal events thought to be related to PDB.

Biochemical markers

For peer review only - http://bmjopen.bmj.com/site/about/guidelines.xhtml

Measurements of serum creatinine, urea and electrolytes, serum total alkaline phosphatase, serum calcium, albumin and liver function tests (AST and/or ALT, γ–GT, bilirubin) and full blood count will be performed using standard techniques at the local laboratories in participating centres. Estimated GFR (eGFR) will be calculated from serum creatinine, gender and weight by the Cockcroft-Gault equation (15). Specialised biochemical markers of bone turnover will be measured centrally at the University of East Anglia. These will include urine N-telopeptide collagen cross links (NTX) corrected for urinary creatinine; serum c-terminal collagen cross-links (CTX), bone-specific alkaline phosphatase (BSAP) and the procollagen type-I N-terminal propeptide fragment (PINP). These measurements will be made on fasting samples collected between 09.00-12.00. Urine samples will be second-voided "spot" samples collected after an overnight fast. Additional biomarkers of bone metabolism may also be assessed if new information indicates that these may be of interest as the study progresses. The serum, plasma and urine samples will be aliquoted and stored locally at -80°C and shipped on dry ice to the central laboratory.

Health related quality of life

Health-related quality of life will be assessed by completion of the SF36 questionnaire (16) at baseline, annual visits and the end of study visit.

Pain

The presence and location of pain will be assessed by completion of the Brief Pain Inventory (BPI) (17) at baseline, annual visits and the end of study visit. Participants will also be asked if they have experienced any pain and bone pain

Anxiety and depression

Anxiety and depression will be assessed by the Hospital Anxiety and Depression Questionnaire (HADS) (18).

Paget's disease related skeletal events

Participants will be evaluated clinically at the end of study for the presence of Paget's disease-related skeletal events (PDRSE). These will include pathological fractures, bone deformity, deafness due to skull involvement, and joint replacement surgery or other surgical procedures that are a carried out because of PDB. Administration of an antiresorptive drug during the study because of signs or symptoms that are thought to be due to PDB will be considered as a PDRSE as will the development of new bone lesions on bone scan. All events will combined for each treatment group to give a total score.

Genetic testing

For peer review only

Genetic testing will be conducted to determine eligibility by Sanger sequencing of exons 7 and 8 of *SQSTM1* and the intron-exon boundaries using DNA extracted from a venous blood sample according to standard techniques (7).

Sample size

The sample size was chosen assuming that 15% of patients in the placebo group and 1.5% of patients in the active (ZA) treatment group will develop new PDB-like bone lesions during follow up. This was based on the observation that ZA has been reported to normalise biochemical markers of bone turnover for up to 6.5 years in 90% of patients with established PDB (19). With this assumption, 85 subjects in each group would provide 89% power to detect a treatment effect of this magnitude at an alpha of 0.05. Since it is possible that more than one affected subject per family could be enrolled, the sample size was inflated to account for relatedness of individuals. This was done by calculating the mean squared alkaline phosphatase values in patients within families who carried the same mutation (271.3) and the mean squared alkaline phosphatase values between families (619.7) and combining this with the estimated average number of two subjects per family who may be enrolled in the study. This resulted in a design effect factor of 1.39, inflating the required sample size to 118 per group. In addition to this the sample size was further inflated to account for a 10% rate of participants lost to follow up resulting in a total sample size of 130 subjects per group or 260 subjects in total. The actual number of subjects randomised to the interventional study by the time recruitment had closed in April 2015 was 222 and to the observational study was 135. The decision to stop recruitment was based on funding, and justified by recalculating the design factor based on the actual number of subjects per family that had been enrolled into the study (1.5 on average). The design factor was recalculated to be 1.26.

Methodology

Eligibility

Those eligible will be 30 years of age or older, with a positive family history of PDB, in whom genetic testing had shown a pathogenic mutation in *SQSTM1.* Individuals who had already been diagnosed with PDB prior to the baseline visit were excluded, as were those with contraindications to ZA as summarised in Table 3. In order to identify people who may be eligible for participation, an extensive programme of genetic testing of probands for *SQSTM1* mutations was carried out. An overview of this process is summarised in Figure 1. A total of 2398 patients with a diagnosis of PDB (probands) identified through various sources were contacted by letter and asked if they would like to be tested for the presence of mutations in the *SQSTM1* gene and 1451 agreed (60.5%). Those that tested negative for *SQSTM1* mutations (n=1203; 82.9%) were informed of the result and counselled but they and their family members were excluded from further involvement in the study. Those that tested

For peer review only - http://bmjopen.bmj.com/site/about/guidelines.xhtml

positive (n=248; 17.1%) were informed of the result, counselled about the implications and asked to pass an information pack about the trial onto any eligible blood relatives with a reply slip that could be returned to the local recruiting centre. Subsequently, a programme of genetic testing for *SQSTM1* mutations was conducted on relatives to identify individuals who may be eligible to take part in the trial. The results of this process are summarised in Figure 2. Information was passed onto 1310 relatives about the study and 751 (57.3%) agreed to be tested. As the result of this, we identified 351 individuals (46.7%) who tested positive and of those, 222 (63.2%) consented to take part in the trial.. Participants with serum 25(OH) vitamin D levels below the lower limit of the local reference range were permitted to take part in the trial but only after they had been treated with vitamin D supplements in order to mitigate the risk of hypocalcaemia following ZA treatment. Recruitment into the clinical trial was also delayed in participants who were scheduled to have dental surgery (tooth extractions, root treatment, or other surgery to the mandible or maxilla), until healing had occurred to mitigate the risk of osteonecrosis of the jaw. Likewise, if the potential participant had dental surgery planned within the first 3 months of the expected infusion date, their recruitment was delayed until healing was complete. Minor dental procedures such as de-scaling and fillings did not constitute a barrier to enrolment. Participants in the Republic of Ireland were required to undergo a dental examination within 1 month prior to the baseline visit at the request of Health Products Regulatory Authority in the Republic of Ireland. Women who were pregnant or breastfeeding were excluded. Women of childbearing potential were permitted to take part provided that they used a robust form of contraception before and for at least 12 months after the ZA infusion.

Observational study

During the genetic testing phase we identified 400 (53.3%) who tested negative for *SQSTM1* mutations and 135 (33.7%) agreed to enter an observational study. Participants in the observational study will have health related quality of life and anxiety and depression assessed by completion of the SF36 and HADS questionnaires at the baseline and end of study visits. They also will have samples for routine biochemistry checked at baseline and the end of study visit and will have samples stored for assessment of biochemical markers of bone turnover.

Consent

The consent process was divided into three stages. The first phase involved obtaining consent from patients with PDB (probands). The second phase involved obtaining consent from relatives (almost exclusively children) of probands for genetic testing. Although the relatives were made aware of the trial, the consent was obtained only for genetic testing, without any commitment to enter the trial. The third phase involved obtaining consent for entry into the trial or observational study.

For peer review only - http://bmjopen.bmj.com/site/about/guidelines.xhtml

Randomisation

Randomisation was performed by a web based tool hosted by Edinburgh Clinical Trials Unit (ECTU) which ensured allocation concealment prior to enrolment. The randomisation algorithm used minimisation to ensure that the groups were balanced for prognostic variables thought to influence the occurrence of PDB including; the type of mutation (missense versus truncating or frameshift); gender; whether the baseline radionuclide bone scan had shown lesions suggestive of PDB; whether serum alkaline phosphatase levels at baseline were elevated (yes/no); and by age band: 30-40, 41-50, 51-60, 61-70, and 71 or over. Following randomisation, the study database generated a treatment code which was used by the research pharmacies in each participating centre to ensure that the correct medication was dispensed.

Pre and post-randomisation withdrawals

Participants will be advised that they have the right to withdraw from the study at any time for any reason. The Investigator will have the right to withdraw a participant at any time if it is deemed to be in the participant's best interest. If a participant decides that they no longer wish to continue with routine assessments or adhere to the study protocol before the planned end of trial assessment, they will be given the opportunity to attend for the end of trial assessment. The same will apply to participants in whom the local investigator decides that adherence to the trial protocol would be inappropriate.

Blinding

The participants and investigators will be blinded to treatment allocation. The ZA and placebo infusions will be identical. Breaking the blind will only be performed where knowledge of the treatment is absolutely necessary for further management of the patient and can only be performed by contacting the local pharmacy, who will have restricted code break details

Interventions

The investigational medicinal product, Zoledronic acid (Aclasta®, Novartis Pharmaceuticals UK Limited, Surrey, United Kingdom), in the treatment of both osteoporosis and Paget's disease of bone (10). The most common side effects are transient flu like symptoms occurring in up to 50% of patients although these are usually mild (20) . The investigational medicinal product was given by intravenous infusion and comprised zoledronic acid (5mg in 100ml ready-to-infuse solution) or a matching placebo. Both will be given at a constant infusion rate over not less than 15 minutes. Medications required for the participants clinical care will be permitted during the study. Should a participant require to be treated with a bone active antiresorptive medication (such as a bisphosphonate, strontium ranelate or denosumab) after randomisation but prior to infusion of IMP, then the participant would not receive study IMP but would still be followed up as per protocol.

For peer review only - http://bmjopen.bmj.com/site/about/guidelines.xhtml

Female patients of child bearing potential will be required to have a negative pregnancy test on the day of, or the day before, the infusion of study drug. Participants are were sexually active will receive specific advice about the possible risks associated with getting pregnant whilst on the trial and will be asked to agree to practice a medically acceptable form of birth control for at least 12 months post-infusion of IMP. Female participants will be excluded from undergo isotope bone scanning during pregnancy or while they are breastfeeding.

Data management

Paper case record forms (CRF) will be provided to record baseline and follow-up clinical measurements and demographics by local research teams. Data from these CRF will be then entered onto a web-based electronic CRF. The Principal Investigator at each study site will be responsible for the quality of the data recorded in the CRF. The ZiPP study eCRF web portal was built and maintained by the software development team of the University of Edinburgh's Clinical Trials Unit, following internal standard operating procedures. A Microsoft stack was used. The back-end repository was MS SQL-Server. The front-end user interface was implemented using ASP.Net technologies.

Adverse event management

Participants will be provided with an event diary to record details of primary care visits, medications taken, hospitalisations and any other adverse effects or health problems. In the event of hospitalisation, the patient will be asked to contact the Principal Investigator (PI) at their local study centre. Adverse events (AE), serious adverse events (SAE) and suspected unexpected serious adverse reactions (SUSAR) will be collected continuously throughout the trial. In addition, participants will be contacted by local research teams one week after receipt of the infusion to record symptoms or side-effects related to this intervention. All adverse events will be recorded from the time a participant consents to join the study until the last study visit has been completed. The investigator or a delegated member of the study team will record adverse events at every visit and participants will be instructed to contact the investigator at any time if adverse events develop. If an AE/SAE occurs, it is the responsibility of the investigator to review all documentation related to the event and evaluate seriousness, causality, severity and expectedness. Events that are considered serious, possibly, probably or definitely related to the investigational medicinal product (serious adverse reactions, SAR) and unexpected (SUSAR) may be unblinded if it is necessary for clinical care. Once the investigator becomes aware that an SAE has occurred, they must report the information to the Clinical Research Governance & quality assurance office of the sponsor within 24 hours. The investigator will then be required to complete a Serious Adverse Event (SAE) form to assess causality, seriousness, severity and expectedness of the event.

For peer review only - http://bmjopen.bmj.com/site/about/guidelines.xhtml

ANALYSIS

Statistical analysis

The principal analysis will be conducted on an intention-to-treat basis. All analyses will allow for clustering by family, and all primary analyses will be adjusted for the minimisation variables. Comparisons will be performed using an appropriate linear modelling procedure, taking into account repeated measures where these are available. Patients with completely missing data for a particular outcome will be removed from the analysis of that particular outcome. The effect of this will be examined using sensitivity analysis. Other sensitivity analyses will look at unadjusted analyses, and the effect of adjusting for centre.

Trial Oversight

Monitoring will be performed in accordance with a study monitoring plan developed by the trial's sponsor. The Principal Investigators and institutions involved in the study will permit trial related monitoring, audits, Research Ethics Committee review, and regulatory inspection(s). A Trial Steering Committee (TSC) will be established to oversee the conduct and progress of the trial. An independent Data Monitoring Committee will be established to oversee the safety of subjects in the trial. The study is expected to provide new information on the evolution of PDB in this participant group as well to give an indication whether zoledronic acid treatment can modify the natural history of the disease. Given the relatively short time frame it's unlikely that the trial will demonstrate any clinical benefits of the treatment in terms of complications of PDB such as pain, fractures, deafness or bone deformity, but patients will be evaluated clinically for the presence of any of these complications should they occur.

Patient and public involvement

The study was designed with the involvement of patients and the Paget's Association – a patient support group. The trial steering committee included a representative of the Paget's Association and a patient representative.

ETHICS AND DISSEMINATION

Ethical approval was granted by the Fife and Forth Valley Research Ethics Committee on 22nd December 2008 (reference number: 08/S0501/84). The study was also approved by local research ethics committees of all participating centres outside the UK and the medicines regulatory agencies in all participating countries. Written informed consent was provided by all participants. The results of the study will be submitted to a peer-reviewed journal so that they are disseminated to the wider medical community. The results will also be disseminated to patients with PDB and their families through the website of the Paget's Association. Authorship on the main paper will be determined by the International Committee of Medical Journal Editors (ICMJE) guidelines. The results of the ZiPP

For peer review only

trial are expected to inform clinical practice and influence clinical guidelines for PDB by determining if early intervention with ZA in pre-symptomatic individuals with *SQSTM1* mutations can prevent or slow the development of bone lesions with an adverse event profile that is acceptable.

Data Sharing

The datasets generated and analysed during this clinical trial are not yet publicly available since data collection is incomplete. It is anticipated that an anonymised dataset will be made available for sharing following completion of the study, database lock and analysis of the primary data.

Funding

The study was funded by the Medical Research Council (UK) (Reference number 85281) and in part by Arthritis Research UK (Reference number 18163). The zoledronic acid and placebo infusions were kindly donated by Novartis Pharmaceuticals.

Authors contributions:

OC and SHR: first draft of the manuscript. Study concept and design: SHR. Obtaining funding: SHR MP, and WDF. Trial management during study set up and recruitment: LF, KG and SHR. Genetic analysis and training of research staff in genetic counselling: MP and RC: Development of statistical analysis plan and sample size calculations: SL and CK, design and maintenance of study database: AW. Participant recruitment and study assessments: SHR, LF, KG, LRR, PLS, GH, RC, SH, JHT, SY-M, MM, RC, WDF, LG, RN, MLB, JdP-M, J-PD, AD, GI, MdiS, NG, JB, MJS, JW, MAK, GCN, ED, GM, AH, NG. Supervision of conduct of the trial. SHR, LF, KG, LRR, PLS, GH, RC, SH, JHT, SY-M, MM, RC, WDF, LG, RN, MLB, JdP-M, J-PD, AD, GI, MdiS, NG, JB, MJS, JW, MAK, GCN, ED, GM, AH, NG, MB, GDM, KC, DW, and RGGR. All authors commented on and revised the manuscript for intellectual content and approved the final version of the manuscript.

Consent for publication:

The manuscript does not contain individual patient data.

Competing interests:

The authors declare that they have no competing interests.

Acknowledgements:

The authors wish to acknowledge the valuable support of the UK Paget's Association in publicising and supporting the study and the many patients with Paget's disease and relatives who supported the study. The authors also wish to acknowledge the contribution of the many research nurses in study centres, Ms Lyndsay Milne from ECTU for data management support, and the laboratory staff in the South East Scotland Genetics Service for conducting mutation analysis of the *SQSTM1* gene.

For peer review only - http://bmjopen.bmj.com/site/about/guidelines.xhtml

Figure legends

Figure 1. Genetic testing phase of the ZIPP study for probands

The figure provides an overview of the process and procedures for genetic testing of probands and contact of family members.

Figure 2. Genetic testing phase for relatives and subsequent enrollment to the ZIPP study.

The figure provides an overview of the process and procedures for genetic testing of relatives as well as an outline of the flow of subjects who consented to participate in the intervention and observational studies.

For peer review only

Table 1. Outcome measures for the ZiPP trial

Primary

- The total number of subjects who develop new bone lesions on radionuclide bone scans with the characteristics of PDB between the baseline visit and the final follow up visit.

Secondary

- The number of new bone lesions on radionuclide bone scan.
- Change in activity of existing bone lesions that were present at baseline assessed by semiquantitative analysis of radionuclide bone scans (21).
- The development of PDB-related skeletal events (PRSE) defined as any one of the following:
  - Development of new bone lesions thought to be due to PDB on imaging
  - Development of complications thought to be due to the development or progression of PDB including pathological fractures, bone deformity, deafness, joint replacement surgery or other orthopaedic procedures
  - Administration of treatment for PDB with an anti-resorptive drug because of the development of signs or symptoms thought to be due to PDB such as pain localised to an affected site or neurological symptoms
- The development of increased bone turnover as assessed by measurement of biochemical markers of bone resorption and bone formation.
- Quality of life, anxiety and depression assessed by the Short Form (36) Health Survey (SF-36), Brief Pain Inventory (BPI), and HADS questionnaires.
- Location, presence and severity of pain assessed by the BPI manikin and pain questionnaire.

For peer review only

Table 2. Summary of assessments and outcome measures for the ZiPP trial.

	Screening	Baseline visit	+1 week	Annual Review	End of study
Medical History		✓		✓	✓
Current medication		✓		✓	✓
Physical Examination		✓			
Height, weight, blood pressure		✓			✓
Routine Biochemistry[1]	✓	✓		✓	✓
Haematology[2]		✓			✓
Blood for Biomarkers[3]		✓		✓	✓
Urine for Biomarkers[4]		✓			✓
SQSTM1 genotyping	✓				
25(OH) vitamin D	✓				
Pregnancy Test[5] (in women of child-bearing potential)		✓			
Isotope Bone Scan		✓			✓
Radiographs or other imaging[6]		✓			✓
Infusion		✓			
Telephone Questionnaire			✓		
Food Frequency Questionnaire		✓			
SF36, HADS, & BPI questionnaires		✓		✓	✓
PDB-related skeletal events					✓

[1]. – Calcium, albumin/total protein, alkaline phosphatase, liver function (AST, ALT, GGT, bilirubin), urea and electrolytes & creatinine (U&E),. [2] – Full blood count . [3] - Blood samples for measurement of bone specific alkaline phosphatase (BSAP), and other specialised markers of bone metabolism. [4] – Second-voided morning urine to be taken and stored for measurement of N-telopeptide collagen cross links (NTX), deoxypyridinoline/creatinine ratio (DPD) and other specialised markers of bone metabolism. [5] - A negative pregnancy test must be obtained on the day of, or the day before, infusion of the study drug. The preferred method of is serum beta-hCG, but a urine beta-hCG is acceptable for centres that are unable to obtain a serum beta-hCG. [6] – To be taken of relevant areas in subjects suspected to have PDB-like bone lesions on bone scan.

For peer review only

Table 3. Eligibility and exclusion criteria for the ZiPP trial

Eligibility criteria
Carrier of *SQSTM1* mutation
Aged 30 years or older
Not already diagnosed with PDB
Exclusion criteria
Already diagnosed with PDB
Unwilling or unable to provide informed consent
Contraindication to bisphosphonates
Estimated GFR (eGFR) < 35ml/min[1]
Hypocalcaemia
Receiving bisphosphonate therapy for another reason
Osteonecrosis of the jaw (ONJ)
Metastatic cancer or cancer diagnosed less than 2 years ago where treatment is still ongoing
Active uveitis, iritis, or episcleritis
Already taking part in another randomised controlled clinical trial
Pregnancy or lactation

For peer review only

For peer review only

For peer review only - http://bmjopen.bmj.com/site/about/guidelines.xhtml

References

1. Laurin N, Brown JP, Morissette J, Raymond V. Recurrent Mutation of the Gene Encoding sequestosome 1 (SQSTM1/p62) in Paget Disease of Bone. Am J Hum Genet. 2002;70(6):1582-8.

2. Barton A, Thomson W, Ke X, Eyre S, Hinks A, Bowes J, et al. Rheumatoid arthritis susceptibility loci at chromosomes 10p15, 12q13 and 22q13. Nat Genet. 2008;40(10):1156-9.

3. Hocking LJ, Lucas GJA, Daroszewska A, Cundy T, Nicholson GC, Donath J, et al. Novel UBA domain mutations of SQSTM1 in Paget's disease of bone: genotype phenotype correlation, functional analysis and structural consequences. J Bone Miner Res. 2004;19(7):1122-7.

4. Hocking LJ, Lucas GJA, Daroszewska A, Mangion J, Olavesen M, Nicholson GC, et al. Domain specific mutations in Sequestosome 1 (SQSTM1) cause familial and sporadic Paget's disease. Hum Mol Genet. 2002;11(22):2735-9.

5. Eekhoff EW, Karperien M, Houtsma D, Zwinderman AH, Dragoiescu C, Kneppers AL, et al. Familial Paget's disease in The Netherlands: occurrence, identification of new mutations in the sequestosome 1 gene, and their clinical associations. Arth Rheum. 2004;50(5):1650-4.

6. Gennari L, Gianfrancesco F, Di Stefano M, Rendina D, Merlotti D, Esposito T, et al. SQSTM1 gene analysis and gene-environment interaction in Paget's disease of bone. J Bone Miner Res. 2010;25(6):1375-84.

7. Visconti MR, Langston AL, Alonso N, Goodman K, Selby PL, Fraser WD, et al. Mutations of SQSTM1 are associated with severity and clinical outcome in Paget's disease of bone. J Bone Miner Res. 2010;25(11):2368-73.

8. Morissette J, Laurin N, Brown JP. Sequestosome 1: Mutation Frequencies, Haplotypes, and Phenotypes in Familial Paget's Disease of Bone. J Bone Miner Res. 2006;21 Suppl 2:38-44.

9. Bolland MJ, Tong PC, Naot D, Callon KE, Wattie DJ, Gamble GD, et al. Delayed Development of Paget's Disease in Offspring Inheriting SQSTM1 Mutations. J Bone Miner Res. 2007;22(3):411-5.

10. Corral-Gudino L, Tan AJ, Del Pino-Montes J, Ralston SH. Bisphosphonates for Paget's disease of bone in adults. Cochrane Database Syst Rev. 2017;12:CD004956.

11. Reid IR, Lyles K, Su G, Brown JP, Walsh JP, del Pino-Montes J, et al. A single infusion of zoledronic acid produces sustained remissions in Paget disease: data to 6.5 years. J Bone Miner Res. 2011;26(9):2261-70.

12. Ralston SH, Corral-Gudino L, Cooper C, Francis RM, Fraser WD, Gennari L, et al. Diagnosis and Management of Paget's Disease of Bone in Adults: A Clinical Guideline. J Bone Miner Res. 2019:e3657.

For peer review only

13. Langston AL, Campbell MK, Fraser WD, MacLennan GS, Selby PL, Ralston SH. Randomised Trial of Intensive Bisphosphonate Treatment Versus Symptomatic Management in Paget's Disease of Bone. J Bone Miner Res. 2010;25:20-31.

14. Tan A, Goodman K, Walker A, Hudson J, MacLennan GS, Selby PL, et al. Long-Term Randomized Trial of Intensive Versus Symptomatic Management in Paget's Disease of Bone: The PRISM-EZ Study. J Bone Miner Res. 2017;32(6):1165-73.

15. Cockcroft DW, Gault MH. Prediction of creatinine clearance from serum creatinine. Nephron. 1976;16(1):31-41.

16. Ware JE, Jr., Gandek B. Overview of the SF-36 Health Survey and the International Quality of Life Assessment (IQOLA) Project. J Clin Epidemiol. 1998;51(11):903-12.

17. Atkinson TM, Mendoza TR, Sit L, Passik S, Scher HI, Cleeland C, et al. The Brief Pain Inventory and its "pain at its worst in the last 24 hours" item: clinical trial endpoint considerations. Pain Med. 2010;11(3):337-46.

18. Crawford JR, Henry JD, Crombie C, Taylor EP. Normative data for the HADS from a large non-clinical sample. Br J Clin Psychol. 2001;40(Pt 4):429-34.

19. Reid IR, Miller P, Lyles K, Fraser W, Brown JP, Saidi Y, et al. Comparison of a single infusion of zoledronic acid with risedronate for Paget's disease. N Engl J Med. 2005;353(9):898-908.

20. Reid IR, Gamble GD, Mesenbrink P, Lakatos P, Black DM. Characterization of and risk factors for the acute-phase response after zoledronic acid. J Clin Endocrinol Metab. 2010;95(9):4380-7.

21. Patel S, Pearson D, Hosking DJ. Quantitative bone scintigraphy in the management of monostotic Paget's disease of bone. Arthritis Rheum. 1995;38(10):1506-12.

For peer review only

For peer review only - http://bmjopen.bmj.com/site/about/guidelines.xhtml

For peer review only

For peer review only - http://bmjopen.bmj.com/site/about/guidelines.xhtml

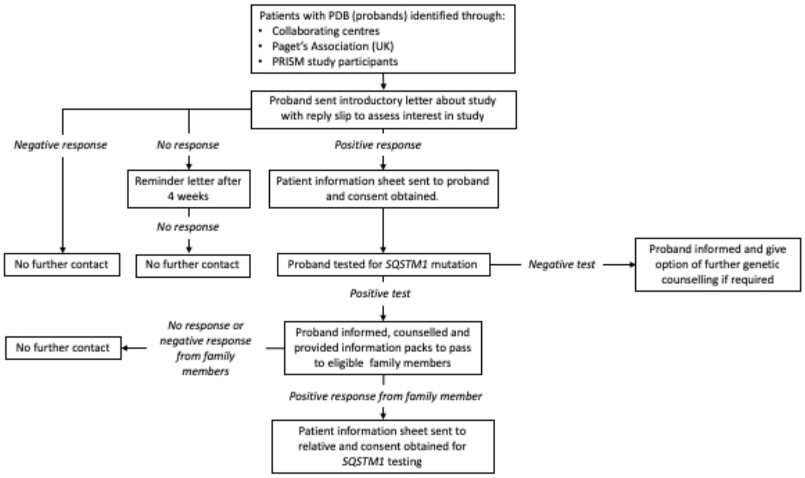

Figure 1

338x190mm (54 x 54 DPI)

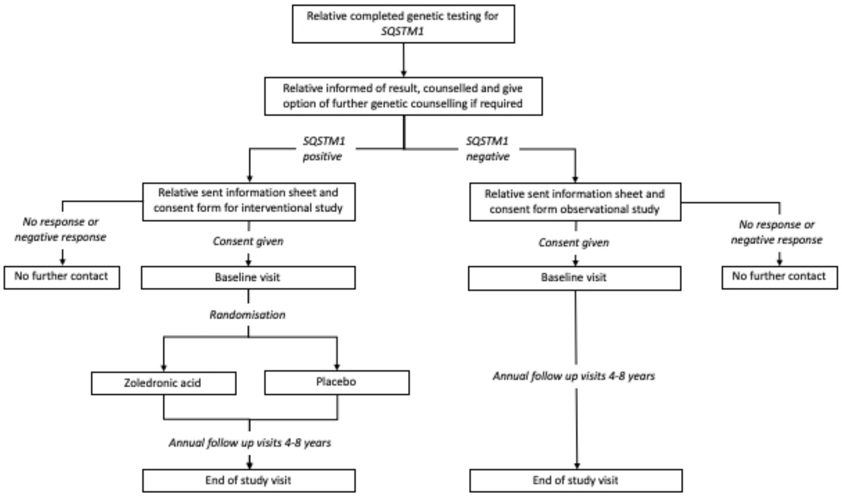

Figure 2

338x190mm (54 x 54 DPI)

STANDARD PROTOCOL ITEMS: RECOMMENDATIONS FOR INTERVENTIONAL TRIALS

SPIRIT 2013 Checklist: Recommended items to address in a clinical trial protocol and related documents*

Section/item	Item No	Description	Page
**Administrative information**			
Title	1	Descriptive title identifying the study design, population, interventions, and, if applicable, trial acronym	1
Trial registration	2a	Trial identifier and registry name. If not yet registered, name of intended registry	3
	2b	All items from the World Health Organization Trial Registration Data Set	N/A
Protocol version	3	Date and version identifier	13
Funding	4	Sources and types of financial, material, and other support	14
Roles and responsibilities	5a	Names, affiliations, and roles of protocol contributors	2, 13
	5b	Name and contact information for the trial sponsor	13
	5c	Role of study sponsor and funders, if any, in study design; collection, management, analysis, and interpretation of data; writing of the report; and the decision to submit the report for publication, including whether they will have ultimate authority over any of these activities	13
	5d	Composition, roles, and responsibilities of the coordinating centre, steering committee, endpoint adjudication committee, data management team, and other individuals or groups overseeing the trial, if applicable (see Item 21a for data monitoring committee)	11
**Introduction**			
Background and rationale	6a	Description of research question and justification for undertaking the trial, including summary of relevant studies (published and unpublished) examining benefits and harms for each intervention	5
	6b	Explanation for choice of comparators	5
Objectives	7	Specific objectives or hypotheses	6

For peer review only

Trial design	8	Description of trial design including type of trial (eg, parallel group, crossover, factorial, single group), allocation ratio, and framework (eg, superiority, equivalence, noninferiority, exploratory)	6
**Methods: Participants, interventions, and outcomes**			
Study setting	9	Description of study settings (eg, community clinic, academic hospital) and list of countries where data will be collected. Reference to where list of study sites can be obtained	7
Eligibility criteria	10	Inclusion and exclusion criteria for participants. If applicable, eligibility criteria for study centres and individuals who will perform the interventions (eg, surgeons, psychotherapists)	7
Interventions	11a	Interventions for each group with sufficient detail to allow replication, including how and when they will be administered	8,9
	11b	Criteria for discontinuing or modifying allocated interventions for a given trial participant (eg, drug dose change in response to harms, participant request, or improving/worsening disease)	N/A
	11c	Strategies to improve adherence to intervention protocols, and any procedures for monitoring adherence (eg, drug tablet return, laboratory tests)	N/A
	11d	Relevant concomitant care and interventions that are permitted or prohibited during the trial	10
Outcomes	12	Primary, secondary, and other outcomes, including the specific measurement variable (eg, systolic blood pressure), analysis metric (eg, change from baseline, final value, time to event), method of aggregation (eg, median, proportion), and time point for each outcome. Explanation of the clinical relevance of chosen efficacy and harm outcomes is strongly recommended	11
Participant timeline	13	Time schedule of enrolment, interventions (including any run-ins and washouts), assessments, and visits for participants. A schematic diagram is highly recommended (see Figure)	24, 25 (Figures 1 & 2)
Sample size	14	Estimated number of participants needed to achieve study objectives and how it was determined, including clinical and statistical assumptions supporting any sample size calculations	11, 12

For peer review only - http://bmjopen.bmj.com/site/about/guidelines.xhtml

Recruitment	15	Strategies for achieving adequate participant enrolment to reach target sample size	12
**Methods: Assignment of interventions (for controlled trials)**			
Allocation:			
Sequence generation	16a	Method of generating the allocation sequence (eg, computer-generated random numbers), and list of any factors for stratification. To reduce predictability of a random sequence, details of any planned restriction (eg, blocking) should be provided in a separate document that is unavailable to those who enrol participants or assign interventions	8
Allocation concealment mechanism	16b	Mechanism of implementing the allocation sequence (eg, central telephone; sequentially numbered, opaque, sealed envelopes), describing any steps to conceal the sequence until interventions are assigned	8
Implementation	16c	Who will generate the allocation sequence, who will enrol participants, and who will assign participants to interventions	8
Blinding (masking)	17a	Who will be blinded after assignment to interventions (eg, trial participants, care providers, outcome assessors, data analysts), and how	8
	17b	If blinded, circumstances under which unblinding is permissible, and procedure for revealing a participant's allocated intervention during the trial	9
**Methods: Data collection, management, and analysis**			
Data collection methods	18a	Plans for assessment and collection of outcome, baseline, and other trial data, including any related processes to promote data quality (eg, duplicate measurements, training of assessors) and a description of study instruments (e.g. questionnaires, laboratory tests) along with their reliability and validity, if known. Reference to where data collection forms can be found, if not in the protocol	10,11
	18b	Plans to promote participant retention and complete follow-up, including list of any outcome data to be collected for participants who discontinue or deviate from intervention protocols	10
Data management	19	Plans for data entry, coding, security, and storage, including any related processes to promote data quality (eg, double data entry; range checks for data values). Reference to where details of data management procedures can be found, if not in the protocol	10

For peer review only - http://bmjopen.bmj.com/site/about/guidelines.xhtml

Statistical methods	20a	Statistical methods for analysing primary and secondary outcomes. Reference to where other details of the statistical analysis plan can be found, if not in the protocol	12,13
	20b	Methods for any additional analyses (eg, subgroup and adjusted analyses)	12,13
	20c	Definition of analysis population relating to protocol non-adherence (eg, as randomised analysis), and any statistical methods to handle missing data (eg, multiple imputation)	12,13
**Methods: Monitoring**			
Data monitoring	21a	Composition of data monitoring committee (DMC); summary of its role and reporting structure; statement of whether it is independent from the sponsor and competing interests; and reference to where further details about its charter can be found, if not in the protocol. Alternatively, an explanation of why a DMC is not needed	12
	21b	Description of any interim analyses and stopping guidelines, including who will have access to these interim results and make the final decision to terminate the trial	N/A
Harms	22	Plans for collecting, assessing, reporting, and managing solicited and spontaneously reported adverse events and other unintended effects of trial interventions or trial conduct	13
Auditing	23	Frequency and procedures for auditing trial conduct, if any, and whether the process will be independent from investigators and the sponsor	12
**Ethics and dissemination**			
Research ethics approval	24	Plans for seeking research ethics committee/institutional review board (REC/IRB) approval	15
Protocol amendments	25	Plans for communicating important protocol modifications (eg, changes to eligibility criteria, outcomes, analyses) to relevant parties (eg, investigators, REC/IRBs, trial participants, trial registries, journals, regulators)	N/A
Consent or assent	26a	Who will obtain informed consent or assent from potential trial participants or authorised surrogates, and how (see Item 32)	7
	26b	Additional consent provisions for collection and use of participant data and biological specimens in ancillary studies, if applicable	N/A

For peer review only - http://bmjopen.bmj.com/site/about/guidelines.xhtml

Confidentiality	27	How personal information about potential and enrolled participants will be collected, shared, and maintained in order to protect confidentiality before, during, and after the trial	
Declaration of interests	28	Financial and other competing interests for principal investigators for the overall trial and each study site	
Access to data	29	Statement of who will have access to the final trial dataset, and disclosure of contractual agreements that limit such access for investigators	N/A
Ancillary and post-trial care	30	Provisions, if any, for ancillary and post-trial care, and for compensation to those who suffer harm from trial participation	N/A
Dissemination policy	31a	Plans for investigators and sponsor to communicate trial results to participants, healthcare professionals, the public, and other relevant groups (eg, via publication, reporting in results databases, or other data sharing arrangements), including any publication restrictions	15
	31b	Authorship eligibility guidelines and any intended use of professional writers	15
	31c	Plans, if any, for granting public access to the full protocol, participant-level dataset, and statistical code	15
**Appendices**			
Informed consent materials	32	Model consent form and other related documentation given to participants and authorised surrogates	
Biological specimens	33	Plans for collection, laboratory evaluation, and storage of biological specimens for genetic or molecular analysis in the current trial and for future use in ancillary studies, if applicable	10,11

*It is strongly recommended that this checklist be read in conjunction with the SPIRIT 2013 Explanation & Elaboration for important clarification on the items. Amendments to the protocol should be tracked and dated. The SPIRIT checklist is copyrighted by the SPIRIT Group under the Creative Commons "Attribution-NonCommercial-NoDerivs 3.0 Unported" license.

For peer review only

BMJ Open

Zoledronate in the prevention of Paget's (ZiPP): Protocol for a randomised trial of genetic testing and targeted Zoledronic acid therapy to prevent SQSTM1-mediated Paget's disease of bone.

Journal:	*BMJ Open*
Manuscript ID	bmjopen-2019-030689.R1
Article Type:	Protocol
Date Submitted by the Author:	13-Jul-2019

Complete List of Authors:	Cronin, Owen; University College Cork National University of Ireland, Department of Medicine; University College Cork APC Microbiome Institute,<br>Forsyth, Laura; University of Edinburgh College of Medicine and Veterinary Medicine<br>Goodman, Kirsteen; Glasgow Caledonian University School of Nursing Midwifery and Community Health<br>Lewis, Steff; University of Edinburgh, Public Health Sciences<br>Keerie, Catriona; University of Edinburgh College of Medicine and Veterinary Medicine<br>Walker, Allan; University of Edinburgh College of Medicine and Veterinary Medicine<br>Porteous, Mary; University of Edinburgh College of Medicine and Veterinary Medicine<br>Cetnarskyj, Roseanne; University of Dundee - Fife Campus<br>Ranganath, Lakshminarayan; University of Liverpool<br>Selby, Peter; Manchester Royal Infirmary<br>Hampson, Geeta; King's College London School of Medical Education<br>Chandra, Rama; King's College Hospital<br>Ho, Shu; Robert Jones and Agnes Hunt Orthopaedic and District Hospital NHS Trust<br>Tobias, Jon ; University of Bristol, Musculoskeletal Research Unit, School of Clinical Sciences<br>Young-Min, Steven; Portsmouth Hospitals NHS Trust<br>McKenna, Malachi; University College Dublin<br>Crowley, Rachel; University College Dublin<br>Fraser, William ; Department of Medicine Norwich Medical School, Faculty of Medicine and Health Sciences, University of East Anglia, Norwich Research Park,<br>Gennari, Luigi; University of Siena Faculty of Medicine and Surgery<br>Nuti, Ranuccio; University of Siena Faculty of Medicine and Surgery<br>Brandi, Maria Luisa; University Hospital Careggi<br>Del Pino-Montes, Javier; University Hospital of Salamanca<br>Devogelaer, Jean-Pierre; Cliniques universitaires Saint-Luc<br>DURNEZ, Anne; Cliniques Universitaires Saint-Luc, Rheumatology; Algemeen Ziekenhuis Jan Portaels<br>Isaia, Giancarlo; Università degli Studi di Torino<br>Di Stefano, Marco; Università degli Studi di Torino

	Guanabens, Nuria; University of Barcelona Faculty of Medicine<br>Blanch, Josep; Hospital del Mar<br>Seibel, Markus; University of Sydney, Concord Hospital<br>Walsh, John; Sir Charles Gairdner Hospital, Department of Endocrinology and Diabetes<br>Kotowicz, Mark; Barwon Health, Department of Endocrinology and Diabetes<br>Nicholson, Geoffrey; University of Queensland, Rural Clinical School<br>Duncan, Emma; University of Queensland<br>Major, Gabor ; Royal Newcastle Centre<br>Horne, Anne; University of Auckland, Department of Medicine<br>Gilchrist, Nigel; Princess Margaret Hospital<br>Boers, Maarten; VU University Medical Center, Clinical Epidemiology and Biostatistics<br>Murray, Gordon; University of Edinburgh, Public Health Sciences<br>Charnock, Keith; The Paget's Association<br>Wilkinson, Diana; The Paget's Association<br>Russell, Graham; University of Oxford<br>Ralston, Stuart; University of Edinburgh,

**Primary Subject Heading**:	Rheumatology
Secondary Subject Heading:	Diabetes and endocrinology
Keywords:	Calcium & bone < DIABETES & ENDOCRINOLOGY, GENETICS, RHEUMATOLOGY

SCHOLARONE™ Manuscripts

Zoledronate in the prevention of Paget's (ZiPP): Protocol for a randomised trial of genetic testing and targeted Zoledronic acid therapy to prevent *SQSTM1*-mediated Paget's disease of bone.

Owen Cronin[1], Laura Forsyth[2], Kirsteen Goodman[2 §] Steff Lewis[2], Catriona Keerie[2], Allan Walker[2], Mary Porteous[3], Roseanne Cetnarskyj[3,4], Lakshminarayan R Ranganath[5], Peter L Selby[6], Geeta Hampson[7], Rama Chandra[8], Shu Ho[89], Jon H Tobias[10], Steven Young-Min[11], Malachi McKenna[12,13], Rachel Crowley[12,13], William D. Fraser[14], Luigi Gennari[15], Ranuccio Nuti[15], Maria Luisa Brandi[16], Javier del Pino-Montes[17], Jean-Pierre Devogelaer[18], Anne Durnez[18], Giancarlo Isaia[19], Marco Di Stefano[19], Nuria Guanabens[20], Josep Blanch[21], Markus J Seibel[22], John Walsh[23], Mark A Kotowicz[24], Geoffrey C Nicholson[25], Emma Duncan[26], Gabor Major[27], Anne Horne[28], Nigel Gilchrist[29], Maarten Boers[30], Gordon D Murray[31], Keith Charnock[32], Diana Wilkinson[32], R Graham G Russell[33], Stuart H. Ralston[1,2,34]

1 Rheumatic Diseases Unit, Western General Hospital, Crewe Road South, Edinburgh, EH4 2XU, United Kingdom.
2 Edinburgh Clinical Trials Unit, Usher Institute, University of Edinburgh, Nine Edinburgh Bioquarter, Little France Road, Edinburgh, EH16 4UX, United Kingdom.
3 South East Scotland Molecular Genetics Service, Western General Hospital; Edinburgh EH4 2XY, United Kingdom.
4 School of Nursing & Health Sciences, University of Dundee, Kirkcaldy Campus. Kirkcaldy, KY2 5YS, UK.
5 Department of Clinical Biochemistry and Metabolic Medicine, University of Liverpool, Daulby Street, L69 3GA, United Kingdom.
6 Manchester Royal Infirmary, Oxford Road, Manchester, M13 9WL, United Kingdom.
7 Department of Chemical Pathology and Metabolic Bone Clinic, Department of Rheumatology, St. Thomas' Hospital, Westminster Bridge Road, London, SE1 7EH, United Kingdom.
8 Clinical Biochemistry, King's College Hospital, Denmark Hill, London, SE5 9RS .
9 The Robert Jones and Agnes Hunt Orthopaedic and District Hospital, Twmpath Lane, Oswestry, SY10 7AG, United Kingdom.
10 Musculoskeletal Research Unit, Translational Health Sciences, Bristol Medical School, University of Bristol, Bristol, BS10 5NB, United Kingdom.
11 Department of Rheumatology, Queen Alexandra Hospital, Southwick Hill Road, Cosham, PO6 3LY, United Kingdom.
12 Endocrinology Department, St. Vincent's University Hospital, Elm Park, Dublin 4, Republic of Ireland.
13 University College Dublin, Belfield, Dublin 4, D04 V1W8, Republic of Ireland.
14 Norwich Medical School, Faculty of Medicine and Health Sciences, University of East Anglia, Norwich, NR4 7TJ, United Kingdom.
15 Department of Medicine, Surgery and Neurosciences, University of Sienna, Policlinico Le Scotte, Viale Bracci, Sienna, 53100, Italy.
16 University Hospital of Careggi, Department of Internal Medicine, Viale Peircaccine 6, Florence 50139, Italy.
17 University Hospital of Salamanca, Rheumatology Department, San Vincente 54-132, Salamanca, 370007, Spain.

For peer review only

For peer review only - http://bmjopen.bmj.com/site/about/guidelines.xhtml

[18] Clinique Universitaires Saint-Luc, n-10 Avenue Hippocrate, Brussels, B-1200, Belgium.
[19] Geriatrics and Metabolic Bone Diseases, AOU San Giovanni Battista di Torino Corso, Bramante 88, Torino, 10126, Italy.
[20] Department of Rheumatology, Hospital Clinic, CIBERehd, C/Villarroel 170, University of Barcelona, Barcelona, 8036, Spain.
[21] Hospital del Mar, Passeig Maritim, 08003 Barcelona, Spain.
[22] Bone Research Program, ANZAC Research Institute, and Department of Endocrinology and Metabolism, Concord Repatriation General Hospital, Concord, Sydney, NSW 2139, Australia.
[23] Department of Endocrinology and Diabetes, Sir Charles Gairdner Hospital, Nedlands, WA 6009, Australia and the Medical School, The University of Western Australia, Crawley, Western Australia 6009.
[24] Deakin University, Geelong, Victoria, Australia; Barwon Health, Geelong, Victoria, Australia; and the Department of Medicine-Western Health, Melbourne Medical School, The University of Melbourne, Victoria, Australia.
[25] Rural Clinical School Research Centre, Darlin Heights, the University of Queensland, Queensland 4350, Australia.
[26] Endocrinology Department, Royal Brisbane and Woman's Hospital, Butterfield Street, Herston QLD 4029, Australia.
[27] Rheumatology, Bone and Joint Institute, Royal Newcastle Center, 2 Lookout Road, New Lambton Heights, NSW 2305, Australia.
[28] Department of Medicine, Faculty of Medical and Health Sciences, University of Auckland, Auckland 1142, New Zealand.
[29] The CGM Research Trust, The Princess Margaret Hospital, PO Box 731, Christchurch, New Zealand.
[30] Amsterdam Rheumatology and Immunology Center, ARC, VU University Medical Center, Amsterdam, the Netherlands.
[31] Usher Institute of Population Health Sciences and Informatics, University of Edinburgh, Edinburgh, UK.
[32] The Paget's Association, Moorside Road, Manchester, M27 0EW, UK
[33] Botnar Research Centre, Nuffield Department of Orthopaedics, Rheumatology & Musculoskeletal Sciences, University of Oxford, Oxford, UK and the Mellanby Centre for Bone Research, University of Sheffield, Sheffield, UK
[34] Centre for Genomic and Experimental Medicine, Institute of Genetics and Molecular Medicine, University of Edinburgh, Western General Hospital, Edinburgh, EH 2XU, United Kingdom.

Corresponding author:
Professor Stuart H Ralston
Centre for Genomic and Experimental Medicine,
Institute of Genetics and Molecular Medicine,
University of Edinburgh,
Western General Hospital,
Edinburgh, EH 2XU, United Kingdom.
Email: stuart.ralston@ed.ac.uk

[§] Present address:
NMAHP Research Unit
Glasgow Caledonian University
Cowcaddens Road, Glasgow G4 0BA
UK

For peer review only

ABSTRACT

Introduction: Paget's disease of bone (PDB) is characterised by increased and disorganised bone remodelling affecting one or more skeletal sites. Complications include bone pain, deformity, deafness and pathological fractures. Mutations in *SQSTM1* are strongly associated with development of PDB. Bisphosphonate therapy can improve bone pain in PDB, but there is no evidence that treatment alters the natural history of PDB or prevents complications. The ZiPP trial will determine if prophylactic therapy with the bisphosphonate zoledronic acid (ZA) can delay or prevent the development of PDB in people who carry *SQSTM1* mutations.

Methods and analysis: People with a family history of PDB aged >30 years who test positive for *SQSTM1* mutations are eligible to take part. At the baseline visit participants are screened for the presence of lesions by radionuclide bone scan. Biochemical markers of bone turnover will be measured and questionnaires completed to assess pain, health related quality of life (HRQoL), anxiety and depression. Participants will be randomised to receive a single intravenous infusion of 5mg ZA or placebo and followed up annually for between 4 and 8 years at which point baseline assessments will be repeated. The primary endpoint will be new bone lesions assessed by radionuclide bone scan. Secondary endpoints will include changes in biochemical markers of bone turnover, pain, HRQoL, anxiety, depression and PDB-related skeletal events.

Ethics and Dissemination: The study was approved by the Fife and Forth Valley Research Ethics Committee on 22nd December 2008 (08/S0501/84). Following completion of the trial, a manuscript will be submitted to a peer-reviewed journal. The results of this trial will inform clinical practice by determining if early intervention with ZA in pre-symptomatic individuals with *SQSTM1* mutations can prevent or slow the development of bone lesions with an adverse event profile that is acceptable.

Trial registration number: ISRCTN11616770; Pre-results.

Abstract: 298 words

Total word count: 3567

For peer review only

Strengths and limitations

- This is the first randomised placebo-controlled trial to test whether genetic testing coupled with targeted intervention with zoledronic acid can modify the development and progression of bone lesions secondary to Paget's disease.
- The inclusion of individuals with SQSTM1 mutations ensures that participants have a high risk of developing Paget's disease and provides a suitable cohort in which to study the potential benefit of prophylactic treatment.
- The choice of zoledronic acid maximises the likelihood of prevent the development of bone lesions in this high risk population.
- The randomised double-blind placebo-controlled design of the trial reduces the risk of selection and assessment bias.
- The study duration is unlikely to be sufficient to evaluate the effect of the intervention on complications of Paget's disease such as bone pain, deformity and pathological fractures. Longer periods of follow-up of this cohort will be required.

Keywords:

Paget’s Disease of Bone; bisphosphonate; Zoledronic acid; SQSTM1, genetics; randomized controlled trial.

For peer review only - http://bmjopen.bmj.com/site/about/guidelines.xhtml

BACKGROUND

Paget's disease of bone (PDB) is characterised by areas of increased and disorganised bone turnover affecting one or more skeletal sites. Many affected individuals develop complications such as bone pain, deformity, deafness, pathological fracture and osteoarthritis. Genetic factors play an important role in PDB. Many genetic variants have been identified that predispose to PDB and related syndromes (1, 2) but mutations in sequestosome-1 (*SQSTM1)* are the most important predisposing factor, occurring in up to 50% of patients with a family history of PDB and up to 10% of people who are unaware of having a family history (3-8). Carriers of *SQSTM1* mutations have more severe disease with an earlier age at onset than patients who do not carry mutations (9). Cross-sectional studies indicate that the penetrance of PDB in *SQSTM1* mutation carriers is about 80% above the age of 70 years (10). However, there has been only one prospective study of disease evolution in mutation carriers. This study involved 10 families with *SQSTM1* mutations. 4/23 (17%) of mutation carriers had developed the PDB disease by the age of 50 as assessed by radionuclide bone scanning, but the age at diagnosis was delayed compared with their parents' age (11). Bisphosphonates are considered to be the treatment of choice for PDB, and within the bisphosphonates, zoledronic acid (ZA) is most likely to give a favourable response in terms of bone pain (12). Zoledronic acid has a very long duration of action in patients with established PDB with effects that can last for more than 6 years (13). The main indication for bisphosphonate treatment in PDB is bone pain thought to be due to increased metabolic activity of the disease (12, 14). Although bisphosphonates are highly effective at suppressing elevated bone turnover in PDB, there is no evidence that giving bisphosphonate treatment with the primary aim of suppressing elevated bone turnover is of clinical benefit (14). There is no evidence at present that treatment can prevent complications of PDB such as fracture, bone deformity, deafness or secondary osteoarthritis (15, 16). The Zoledronic acid in the Prevention of Paget's disease (ZiPP) trial has been designed to determine if prophylactic treatment with ZA is effective at preventing the development or progression of bone lesions with the characteristic features of PDB in asymptomatic *SQSTM1* mutation carriers. The reason for choosing people with *SQSTM1* mutations is that these are relatively common in PDB and because carriers of *SQSTM1* mutations have a high risk of developing PDB with increasing age.

Good clinical practice

The study will be carried out according to the principles of the International Conference on Harmonisation Tripartite Guideline for Good Clinical Practice and local guidance and regulations

Consolidated standards of reporting trials

The results of the trial will be reported in accordance with the Consolidated Standards Of Reporting Trials (CONSORT) statement.

For peer review only - http://bmjopen.bmj.com/site/about/guidelines.xhtml

Aim

To improve clinical outcome in PDB by exploring whether genetic testing for *SQSTM1* mutations coupled with prophylactic treatment with ZA, can prevent of slow the development of bone lesions, prevent skeletal complications and favourably modify pain and quality of life in those genetically at risk because of a positive family history.

Objectives

The study objectives are summarised in Table 1 and described in more detail below

The primary objective is:

To quantify the number of subjects in each treatment group who develop new bone lesions with the characteristics of PDB as assessed by radionuclide bone scan. The presence and location of lesions will be assessed by imaging experts blinded to treatment allocation. A new bone lesion will be defined as evidence of involvement of a new bone or part of a previously affected bone at the end-of-study visit which was not thought to be involved at the baseline visit.

The secondary objectives are:

To quantify the number of new bone lesions and change in activity of existing bone lesions present at baseline; to evaluate the effects of treatment on skeletal events related to PDB; to evaluate the effects of treatment on biochemical markers of bone resorption and bone formation; and to evaluate the effects of treatment on health related quality of life, anxiety and depression and the presence, localisation and severity of pain.

Outcome measures

The schedule of assessments and outcome measures which will be collected during the study are summarised in Table 2 and are discussed individually in more detail below.

Bone lesions

These will be assessed by Tc^{99} radionuclide bone scan, which is recognised to be the most sensitive imaging technique for identifying bone lesions in PDB (17). Participants thought to have PDB-like bone lesions on scan may have further imaging performed by x-ray, CT scan or MRI scan if the local investigator considers it clinically indicated. Anonymised bone scan and x-ray images will be uploaded on to the study database for review. All scans will be reviewed by an imaging expert blinded to treatment allocation. A proportion of images will reviewed by a second imaging expert, also blinded to treatment allocation, to evaluate concordance between observers. The images selected will include all of those considered by the primary imaging expert to represent PDB-like lesions. In the event that the experts disagree on a specific image a third imaging expert (also blinded to treatment allocation) will be asked to adjudicate.

Clinical assessments

For peer review only - http://bmjopen.bmj.com/site/about/guidelines.xhtml

Participants will undergo a physical examination at the baseline visit including blood pressure and pulse. Participants will be evaluated clinically at the end of trial visit for any symptoms or signs of skeletal events thought to be related to PDB.

Biochemical markers

Measurements of serum creatinine, urea and electrolytes, serum total alkaline phosphatase, serum calcium, albumin and liver function tests (AST and/or ALT, γ–GT, bilirubin) and full blood count will be performed using standard techniques at the local laboratories in participating centres. Estimated GFR (eGFR) will be calculated from serum creatinine, gender and weight by the Cockcroft-Gault equation (18). Specialised biochemical markers of bone turnover will be measured centrally at the University of East Anglia. These will include urine N-telopeptide collagen cross links (NTX) corrected for urinary creatinine; serum c-terminal collagen cross-links (CTX), bone-specific alkaline phosphatase (BSAP) and the procollagen type-I N-terminal propeptide fragment (PINP). These measurements will be made on fasting samples collected between 09.00-12.00 as previous studies have shown that markers of bone resorption have a circadian rhythm and are influenced by food intake (19). The urine samples will be second-voided "spot" samples collected after an overnight fast. The preferred markers of bone resorption are urinary NTX and serum CTX. These have been found to be elevated in patients with PDB in case control studies and to correlate with the extent of bone lesions as determined by scintigraphy in PDB (20). The preferred markers of bone formation will be PINP and BSAP since both have been shown to be superior to total ALP at detecting PDB in case control studies (17). Additional biomarkers of bone metabolism may also be assessed if new information indicates that these may be of interest as the study progresses. The serum, plasma and urine samples will be aliquoted and stored locally at -80°C and shipped on dry ice to the central laboratory.

Health related quality of life

Health-related quality of life will be assessed by completion of the SF36 questionnaire (21) at baseline, annual visits and the end of study visit. The SF36 is a widely-used, validated questionnaire (21) previously used to assess quality of life in patients with established PDB (15, 16).

Pain

The presence and location of pain will be assessed by completion of the Brief Pain Inventory (BPI) (22) at baseline, annual visits and the end of study visit. The BPI was originally developed to evaluate the location and severity of pain in patients with malignant disease but has since been validated in people with chronic non-malignant pain (23). In addition to completing BPI, participants will also be asked if they have experienced any pain and bone pain

Anxiety and depression

For peer review only - http://bmjopen.bmj.com/site/about/guidelines.xhtml

Anxiety and depression will be assessed by the Hospital Anxiety and Depression Questionnaire (HADS) (24). This questionnaire was chosen since it is quick and simple to administer and has it has been extensively validated in many different countries and settings (25).

Paget's disease related skeletal events

Participants will be evaluated clinically at the end of study for the presence of Paget's disease-related skeletal events (PDRSE). These will include pathological fractures, bone deformity, deafness due to skull involvement, and joint replacement surgery or other surgical procedures that are a carried out because of PDB. Administration of an antiresorptive drug during the study because of signs or symptoms that are thought to be due to PDB will be considered as a PDRSE as will the development of new bone lesions on bone scan. All events will be combined for each treatment group to give a total score.

Genetic testing

Genetic testing will be conducted to determine eligibility by Sanger sequencing of exons 7 and 8 of *SQSTM1* and the intron-exon boundaries using DNA extracted from a venous blood sample according to standard techniques (9).

Sample size

The sample size was chosen assuming that 15% of patients in the placebo group and 1.5% of patients in the active (ZA) treatment group will develop new PDB-like bone lesions during follow-up. This was based on the observation that ZA has been reported to normalise biochemical markers of bone turnover for up to 6.5 years in 90% of patients with established PDB (26). With this assumption, 85 subjects in each group would provide 89% power to detect a treatment effect of this magnitude at an alpha of 0.05. Since it is possible that more than one affected subject per family could be enrolled, the sample size was inflated to account for relatedness of individuals. This was done by calculating the mean squared alkaline phosphatase values in patients within families who carried the same mutation (271.3) and the mean squared alkaline phosphatase values between families (619.7) and combining this with the estimated average number of two subjects per family who may be enrolled in the study. This resulted in a design effect factor of 1.39, inflating the required sample size to 118 per group. In addition to this the sample size was further inflated to account for a 10% rate of participants lost to follow up resulting in a total sample size of 130 subjects per group or 260 subjects in total. The actual number of subjects randomised to the interventional study by the time recruitment had closed in April 2015 was 222 and to the observational study was 135. The decision to stop recruitment was based on funding, and justified by recalculating the design factor based on the actual number of subjects per family that had been enrolled into the study (1.5 on average). The design factor was recalculated to be 1.26.

For peer review only - http://bmjopen.bmj.com/site/about/guidelines.xhtml

Methodology

Eligibility

Those eligible will be 30 years of age or older, with a positive family history of PDB, in whom genetic testing had shown a pathogenic mutation in *SQSTM1.* Individuals who had already been diagnosed with PDB prior to the baseline visit were excluded, as were those with contraindications to ZA as summarised in Table 3. In order to identify people who may be eligible for participation, an extensive programme of genetic testing of probands for *SQSTM1* mutations was carried out. An overview of this process is summarised in Figure 1. Patients with a diagnosis of PDB (probands) identified through various sources were contacted by letter and asked if they would like to be tested for the presence of mutations in the *SQSTM1* gene. Those that tested negative for *SQSTM1* mutations were informed of the result and counselled but they and their family members were excluded from further involvement in the study. Those that tested positive were informed of the result, counselled about the implications and asked to pass an information pack about the trial onto any eligible blood relatives with a reply slip that could be returned to the local recruiting centre. Subsequently, a programme of genetic testing for *SQSTM1* mutations was conducted on relatives to identify individuals who may be eligible to take part in the trial. The results of this process are summarised in Figure 2. Participants with serum 25(OH) vitamin D levels below the lower limit of the local reference range were permitted to take part in the trial but only after they had been treated with vitamin D supplements in order to mitigate the risk of hypocalcaemia following ZA treatment. Recruitment into the clinical trial was also delayed in participants who were scheduled to have dental surgery (tooth extractions, root treatment, or other surgery to the mandible or maxilla), until healing had occurred to mitigate the risk of osteonecrosis of the jaw. Likewise, if the potential participant had dental surgery planned within the first 3 months of the expected infusion date, their recruitment was delayed until healing was complete. Minor dental procedures such as de-scaling and fillings did not constitute a barrier to enrolment. Participants in the Republic of Ireland were required to undergo a dental examination within 1 month prior to the baseline visit at the request of Health Products Regulatory Authority in the Republic of Ireland. Women who were pregnant or breastfeeding were excluded. Women of childbearing potential were permitted to take part provided that they agreed to practice a medically robust form of contraception before and for at least 12 months after the ZA infusion (an intra-uterine device, a barrier method with spermicide, condoms, subdermal implant or oral contraceptive). Women who are pregnant or lactating at the time of randomisation will be excluded. In the event that a woman becomes pregnant or is lactating during the study, bone scanning and x-rays will not be performed until the patient is no longer pregnant and has ceased lactating.

For peer review only - http://bmjopen.bmj.com/site/about/guidelines.xhtml

Observational study

During the genetic testing phase we identified 400 (53.3%) who tested negative for *SQSTM1* mutations and 135 (33.7%) agreed were invited to enrol in an observational study. Participants in the observational study will have health-related quality of life and anxiety and depression measures assessed by completion of the SF36 and HADS questionnaires at the baseline and end of study visits. They also will have samples for routine biochemistry checked at baseline and the end of study visit and will have samples stored for assessment of biochemical markers of bone turnover.

Consent

The consent process was divided into three stages. The first phase involved obtaining consent from patients with PDB (probands). The second phase involved obtaining consent from relatives probands for genetic testing. Although the relatives were made aware of the trial, the consent was obtained only for genetic testing, without any commitment to enter the trial. The third phase involved obtaining consent for entry into the trial or observational study.

Randomisation

Randomisation was performed by a web based tool hosted by Edinburgh Clinical Trials Unit (ECTU) which ensured allocation concealment prior to enrolment. The randomisation algorithm used minimisation to ensure that the groups were balanced for prognostic variables thought to influence the occurrence of PDB including: the type of mutation (missense versus truncating or frameshift); gender; whether the baseline radionuclide bone scan had shown lesions suggestive of PDB; whether serum alkaline phosphatase levels at baseline were elevated (yes/no); and by age band: 30-40, 41-50, 51-60, 61-70, and 71 or over. Following randomisation, the study database generated a treatment code which was used by the research pharmacies in each participating centre to ensure that the correct medication was dispensed.

Pre and post-randomisation withdrawals

Participants will be advised that they have the right to withdraw from the study at any time for any reason. The Investigator will have the right to withdraw a participant at any time if it is deemed to be in the participant's best interest. If a participant decides that they no longer wish to continue with routine assessments or adhere to the study protocol before the planned end of trial assessment, they will be given the opportunity to attend for the end of trial assessment. The same will apply to participants in whom the local investigator decides that adherence to the trial protocol would be inappropriate.

Blinding

The participants and investigators will be blinded to treatment allocation. The ZA and placebo infusions will be identical. Breaking the blind will only be performed where knowledge of the

For peer review only - http://bmjopen.bmj.com/site/about/guidelines.xhtml

treatment is absolutely necessary for further management of the patient and can only be performed by contacting the local pharmacy, who will have restricted code break details.

Interventions

The investigational medicinal product (IMP), Zoledronic acid (Aclasta®, Novartis Pharmaceuticals UK Limited, Surrey, United Kingdom), is used in the treatment of both osteoporosis and Paget's disease of bone (12). The most common side effects are transient flu like symptoms occurring in up to 50% of patients although these are usually mild (27). The IMP was given by intravenous infusion and comprised zoledronic acid (5mg in 100ml ready-to-infuse solution) or a matching placebo. Both will be given at a constant infusion rate over not less than 15 minutes. Medications required for the participants' clinical care will be permitted during the study. Should a participant require treatment with a bone active antiresorptive medication (such as a bisphosphonate, strontium ranelate or denosumab) after randomisation but prior to infusion of the IMP, then the participant would not receive the study IMP but would still be followed up as per protocol. Female patients of child bearing potential will be required to have a negative pregnancy test on the day of, or the day before, the infusion of study drug. Participants who are sexually active will receive specific advice about the possible risks associated with getting pregnant whilst in the trial and will be asked to agree to practice a medically acceptable form of birth control for at least 12 months post-infusion of IMP. Female participants who inadvertently become pregnant during the trial will be excluded from isotope bone scanning during pregnancy or breastfeeding.

Data management

Paper case record forms (CRF) will be provided to record baseline and follow-up clinical measurements and demographics by local research teams. Data from these CRF will then be entered onto a web-based electronic CRF. The Principal Investigator at each study site will be responsible for the quality of the data recorded in the CRF. The ZiPP study eCRF web portal was built and maintained by the software development team of the University of Edinburgh's Clinical Trials Unit, following internal standard operating procedures. A Microsoft stack was used. The back-end repository was MS SQL-Server. The front-end user interface was implemented using ASP.Net technologies.

Adverse event management

Participants will be provided with an event diary to record details of primary care visits, medications taken, hospitalisations and any other adverse effects or health problems. In the event of hospitalisation, the patient will be asked to contact the Principal Investigator (PI) at their local study centre. Adverse events (AE), serious adverse events (SAE) and suspected unexpected serious adverse

For peer review only - http://bmjopen.bmj.com/site/about/guidelines.xhtml

reactions (SUSAR) will be collected continuously throughout the trial. In addition, participants will be contacted by local research teams one week after receipt of the infusion to record symptoms or side-effects related to this intervention. All adverse events will be recorded from the time a participant consents to join the study until the last study visit has been completed. The investigator or a delegated member of the study team will record adverse events at every visit and participants will be instructed to contact the investigator at any time if adverse events develop. If an AE/SAE occurs, it is the responsibility of the investigator to review all documentation related to the event and evaluate seriousness, causality, severity and expectedness. Events that are considered serious, possibly, probably or definitely related to the IMP (serious adverse reactions, SAR) and unexpected (SUSAR) may be unblinded if it is necessary for clinical care. Once the investigator becomes aware that an SAE has occurred, they must report the information to the Clinical Research Governance & quality assurance office of the sponsor within 24 hours. The investigator will then be required to complete a Serious Adverse Event (SAE) form to assess causality, seriousness, severity and expectedness of the event.

ANALYSIS

Statistical analysis

The principal analysis will be conducted on an intention-to-treat basis. All analyses will allow for clustering by family, and all primary analyses will be adjusted for the minimisation of variables. Comparisons will be performed using an appropriate linear modelling procedure, taking into account repeated measures where these are available. Patients with completely missing data for a particular outcome will be removed from the analysis of that particular outcome. The effect of this will be examined using sensitivity analysis. Other sensitivity analyses will look at unadjusted analyses, and the effect of adjusting for centre.

Trial Oversight

Monitoring will be performed in accordance with a study monitoring plan developed by the trial's sponsor. The Principal Investigators and institutions involved in the study will permit trial related monitoring, audits, Research Ethics Committee review, and regulatory inspection(s). A Trial Steering Committee (TSC) will be established to oversee the conduct and progress of the trial. An independent Data Monitoring Committee will be established to oversee the safety of subjects in the trial. The study is expected to provide new information on the evolution of PDB in this participant group as well to give an indication as to whether Zoledronic acid treatment can modify the natural history of the disease. Given the relatively short time frame it's unlikely that the trial will demonstrate any clinical benefits of the treatment in terms of complications of PDB such as pain,

For peer review only - http://bmjopen.bmj.com/site/about/guidelines.xhtml

fractures, deafness or bone deformity, but patients will be evaluated clinically for the presence of any of these complications should they occur.

Patient and public involvement

The study was designed with the involvement of patients and the Paget's Association – a patient support group. The trial steering committee included a representative of the Paget's Association and a patient representative.

ETHICS AND DISSEMINATION

Ethical approval was granted by the Fife and Forth Valley Research Ethics Committee on 22nd December 2008 (reference number: 08/S0501/84). The study was also approved by local research ethics committees of all participating centres outside the UK and the medicines regulatory agencies in all participating countries. Written informed consent was provided by all participants. The results of the study will be submitted to a peer-reviewed journal so that they are disseminated to the wider medical community. The results will also be disseminated to patients with PDB and their families through the website of the Paget's Association. Authorship on the main paper will be determined by the International Committee of Medical Journal Editors (ICMJE) guidelines. The results of the ZiPP trial are expected to inform clinical practice and influence clinical guidelines for PDB by determining if early intervention with ZA in pre-symptomatic individuals with *SQSTM1* mutations can prevent or slow the development of bone lesions with an adverse event profile that is acceptable.

Data Sharing

The datasets generated and analysed during this clinical trial are not yet publicly available since data collection is incomplete. It is anticipated that an anonymised dataset will be made available for sharing following completion of the study, database lock and analysis of the primary data.

Funding

The study was funded by the Medical Research Council (UK) (Reference number 85281) and in part by Arthritis Research UK (Reference number 18163). The zoledronic acid and placebo infusions were kindly donated by Novartis Pharmaceuticals.

Authors contributions:

First draft of the manuscript: OC and SHR; Study concept and design: SHR; Obtaining funding: SHR MP, and WDF; Trial management during study set up and recruitment: LF, KG and SHR; Genetic analysis and training of research staff in genetic counselling: MP and RC; Development of statistical analysis plan and sample size calculations: SL and CK; design and maintenance of study database: AW; Participant recruitment and study assessments: SHR, LF, KG, LRR, PLS, GH, RC, SH, JHT, SY-M, MM, RC, WDF, LG, RN, MLB, JdP-M, J-PD, AD, GI, MdiS, NG, JB, MJS, JW, MAK, GCN, ED, GM, AH, NG; Supervision of conduct of the trial. SHR, LF, KG, LRR, PLS, GH, RC, SH, JHT, SY-M, MM, RC, WDF, LG,

For peer review only - http://bmjopen.bmj.com/site/about/guidelines.xhtml

RN, MLB, JdP-M, J-PD, AD, GI, MdiS, NG, JB, MJS, JW, MAK, GCN, ED, GM, AH, NG, MB, GDM, KC, DW, and RGGR. All authors commented on and revised the manuscript for intellectual content and approved the final version of the manuscript.

Consent for publication:

The manuscript does not contain individual patient data.

Competing interests:

The authors declare that they have no competing interests.

Acknowledgements:

The authors wish to acknowledge the valuable support of the UK Paget's Association in publicising and supporting the study and the many patients with Paget's disease and relatives who supported the study. The authors also wish to acknowledge the contribution of the many research nurses in study centres, Ms Lyndsay Milne from ECTU for data management support, and the laboratory staff in the South East Scotland Genetics Service for conducting mutation analysis of the *SQSTM1* gene.

For peer review only

Figure legends

Figure 1. Genetic testing phase of the ZIPP study for probands

The figure provides an overview of the process and procedures for genetic testing of probands and contact of family members.

Figure 2. Genetic testing phase for relatives and subsequent enrollment to the ZIPP study.

The figure provides an overview of the process and procedures for genetic testing of relatives as well as an outline of the flow of subjects who consented to participate in the intervention and observational studies.

For peer review only - http://bmjopen.bmj.com/site/about/guidelines.xhtml

Table 1. Outcome measures for the ZiPP trial

Primary<br>• The total number of subjects who develop new bone lesions on radionuclide bone scans with the characteristics of PDB between the baseline visit and the final follow up visit.
Secondary<br>• The number of new bone lesions on radionuclide bone scan.<br>• Change in activity of existing bone lesions that were present at baseline assessed by semiquantitative analysis of radionuclide bone scans (28).<br>• The development of PDB-related skeletal events (PRSE) defined as any one of the following:<br>○ Development of new bone lesions thought to be due to PDB on imaging<br>○ Development of complications thought to be due to the development or progression of PDB including pathological fractures, bone deformity, deafness, joint replacement surgery or other orthopaedic procedures<br>○ Administration of treatment for PDB with an anti-resorptive drug because of the development of signs or symptoms thought to be due to PDB such as pain localised to an affected site or neurological symptoms<br>• The development of increased bone turnover as assessed by measurement of biochemical markers of bone resorption and bone formation.<br>• Quality of life, anxiety and depression assessed by the Short Form (36) Health Survey (SF-36), Brief Pain Inventory (BPI), and HADS questionnaires.<br>• Location, presence and severity of pain assessed by the BPI manikin and pain questionnaire.

For peer review only

Table 2. Summary of assessments and outcome measures for the ZiPP trial.

	Screening	Baseline visit	+1 week	Annual Review	End of study
Medical History		✓		✓	✓
Current medication		✓		✓	✓
Physical Examination		✓			
Height, weight, blood pressure		✓			✓
Routine Biochemistry[1]	✓	✓		✓	✓
Haematology[2]		✓			✓
Blood for Biomarkers[3]		✓		✓	✓
Urine for Biomarkers[4]		✓			✓
SQSTM1 genotyping	✓				
25(OH) vitamin D	✓				
Pregnancy Test[5] (in women of child-bearing potential)		✓			
Isotope Bone Scan		✓			✓
Radiographs or other imaging[6]		✓			✓
Infusion		✓			
Telephone Questionnaire			✓		
Food Frequency Questionnaire		✓			
SF36, HADS, & BPI questionnaires		✓		✓	✓
PDB-related skeletal events					✓

[1]. – Calcium, albumin/total protein, alkaline phosphatase, liver function (AST, ALT, GGT, bilirubin), urea and electrolytes & creatinine (U&E),. [2] – Full blood count . [3] - Blood samples for measurement of bone specific alkaline phosphatase (BSAP), and other specialised markers of bone metabolism. [4] – Second-voided morning urine to be taken and stored for measurement of N-telopeptide collagen cross links (NTX), deoxypyridinoline/creatinine ratio (DPD) and other specialised markers of bone metabolism. [5] - A negative pregnancy test must be obtained on the day of, or the day before, infusion of the study drug. The preferred method of is serum beta-hCG, but a urine beta-hCG is acceptable for centres that are unable to obtain a serum beta-hCG. [6] – To be taken of relevant areas in subjects suspected to have PDB-like bone lesions on bone scan.

For peer review only

Table 3. Eligibility and exclusion criteria for the ZiPP trial

Eligibility criteria
Carrier of *SQSTM1* mutation
Aged 30 years or older
Not already diagnosed with PDB
Exclusion criteria
Already diagnosed with PDB
Unwilling or unable to provide informed consent
Contraindication to bisphosphonates
Estimated GFR (eGFR) < 35ml/min[1]
Hypocalcaemia
Receiving bisphosphonate therapy for another reason
Osteonecrosis of the jaw (ONJ)
Metastatic cancer or cancer diagnosed less than 2 years ago where treatment is still ongoing
Active uveitis, iritis, or episcleritis
Already taking part in another randomised controlled clinical trial
Pregnancy or lactation at the time of randomisation or bone scanning

For peer review only

References:

1. Gennari L, Rendina D, Falchetti A, Merlotti D. Paget's Disease of Bone. Calcif Tissue Int. 2019;104(5):483-500.

2. Ralston SH, Taylor JP. Rare Inherited forms of Paget's Disease and Related Syndromes. Calcif Tissue Int. 2019;104(5):501-16.

3. Laurin N, Brown JP, Morissette J, Raymond V. Recurrent Mutation of the Gene Encoding sequestosome 1 (SQSTM1/p62) in Paget Disease of Bone. Am J Hum Genet. 2002;70(6):1582-8.

4. Barton A, Thomson W, Ke X, Eyre S, Hinks A, Bowes J, et al. Rheumatoid arthritis susceptibility loci at chromosomes 10p15, 12q13 and 22q13. Nat Genet. 2008;40(10):1156-9.

5. Hocking LJ, Lucas GJA, Daroszewska A, Cundy T, Nicholson GC, Donath J, et al. Novel UBA domain mutations of SQSTM1 in Paget's disease of bone: genotype phenotype correlation, functional analysis and structural consequences. J Bone Miner Res. 2004;19(7):1122-7.

6. Hocking LJ, Lucas GJA, Daroszewska A, Mangion J, Olavesen M, Nicholson GC, et al. Domain specific mutations in Sequestosome 1 (SQSTM1) cause familial and sporadic Paget's disease. Hum Mol Genet. 2002;11(22):2735-9.

7. Eekhoff EW, Karperien M, Houtsma D, Zwinderman AH, Dragoiescu C, Kneppers AL, et al. Familial Paget's disease in The Netherlands: occurrence, identification of new mutations in the sequestosome 1 gene, and their clinical associations. Arth Rheum. 2004;50(5):1650-4.

8. Gennari L, Gianfrancesco F, Di Stefano M, Rendina D, Merlotti D, Esposito T, et al. SQSTM1 gene analysis and gene-environment interaction in Paget's disease of bone. J Bone Miner Res. 2010;25(6):1375-84.

9. Visconti MR, Langston AL, Alonso N, Goodman K, Selby PL, Fraser WD, et al. Mutations of SQSTM1 are associated with severity and clinical outcome in Paget's disease of bone. J Bone Miner Res. 2010;25(11):2368-73.

10. Morissette J, Laurin N, Brown JP. Sequestosome 1: Mutation Frequencies, Haplotypes, and Phenotypes in Familial Paget's Disease of Bone. J Bone Miner Res. 2006;21 Suppl 2:38-44.

For peer review only - http://bmjopen.bmj.com/site/about/guidelines.xhtml

11. Bolland MJ, Tong PC, Naot D, Callon KE, Wattie DJ, Gamble GD, et al. Delayed Development of Paget's Disease in Offspring Inheriting SQSTM1 Mutations. J Bone Miner Res. 2007;22(3):411-5.

12. Corral-Gudino L, Tan AJ, Del Pino-Montes J, Ralston SH. Bisphosphonates for Paget's disease of bone in adults. Cochrane Database Syst Rev. 2017;12:CD004956.

13. Reid IR, Lyles K, Su G, Brown JP, Walsh JP, del Pino-Montes J, et al. A single infusion of zoledronic acid produces sustained remissions in Paget disease: data to 6.5 years. J Bone Miner Res. 2011;26(9):2261-70.

14. Ralston SH, Corral-Gudino L, Cooper C, Francis RM, Fraser WD, Gennari L, et al. Diagnosis and Management of Paget's Disease of Bone in Adults: A Clinical Guideline. J Bone Miner Res. 2019:e3657.

15. Langston AL, Campbell MK, Fraser WD, MacLennan GS, Selby PL, Ralston SH. Randomised Trial of Intensive Bisphosphonate Treatment Versus Symptomatic Management in Paget's Disease of Bone. J Bone Miner Res. 2010;25:20-31.

16. Tan A, Goodman K, Walker A, Hudson J, MacLennan GS, Selby PL, et al. Long-Term Randomized Trial of Intensive Versus Symptomatic Management in Paget's Disease of Bone: The PRISM-EZ Study. J Bone Miner Res. 2017;32(6):1165-73.

17. Ralston SH, Corral-Gudino L, Cooper C, Francis RM, Fraser WD, Gennari L, et al. Diagnosis and Management of Paget's Disease of Bone in Adults: A Clinical Guideline. J Bone Miner Res. 2019;34(4):579-604.

18. Cockcroft DW, Gault MH. Prediction of creatinine clearance from serum creatinine. Nephron. 1976;16(1):31-41.

19. Greenblatt MB, Tsai JN, Wein MN. Bone Turnover Markers in the Diagnosis and Monitoring of Metabolic Bone Disease. Clin Chem. 2017;63(2):464-74.

20. Al Nofal AA, Altayar O, BenKhadra K, Qasim Agha OQ, Asi N, Nabhan M, et al. Bone turnover markers in Paget's disease of the bone: A Systematic review and meta-analysis. Osteoporos Int. 2015;26(7):1875-91.

For peer review only

For peer review only - http://bmjopen.bmj.com/site/about/guidelines.xhtml

21. Ware JE, Jr., Gandek B. Overview of the SF-36 Health Survey and the International Quality of Life Assessment (IQOLA) Project. J Clin Epidemiol. 1998;51(11):903-12.

22. Atkinson TM, Mendoza TR, Sit L, Passik S, Scher HI, Cleeland C, et al. The Brief Pain Inventory and its "pain at its worst in the last 24 hours" item: clinical trial endpoint considerations. Pain Med. 2010;11(3):337-46.

23. Tan G, Jensen MP, Thornby JI, Shanti BF. Validation of the Brief Pain Inventory for chronic nonmalignant pain. J Pain. 2004;5(2):133-7.

24. Crawford JR, Henry JD, Crombie C, Taylor EP. Normative data for the HADS from a large non-clinical sample. Br J Clin Psychol. 2001;40(Pt 4):429-34.

25. Stern AF. The hospital anxiety and depression scale. Occup Med (Lond). 2014;64(5):393-4.

26. Reid IR, Miller P, Lyles K, Fraser W, Brown JP, Saidi Y, et al. Comparison of a single infusion of zoledronic acid with risedronate for Paget's disease. N Engl J Med. 2005;353(9):898-908.

27. Reid IR, Gamble GD, Mesenbrink P, Lakatos P, Black DM. Characterization of and risk factors for the acute-phase response after zoledronic acid. J Clin Endocrinol Metab. 2010;95(9):4380-7.

28. Patel S, Pearson D, Hosking DJ. Quantitative bone scintigraphy in the management of monostotic Paget's disease of bone. Arthritis Rheum. 1995;38(10):1506-12.

For peer review only

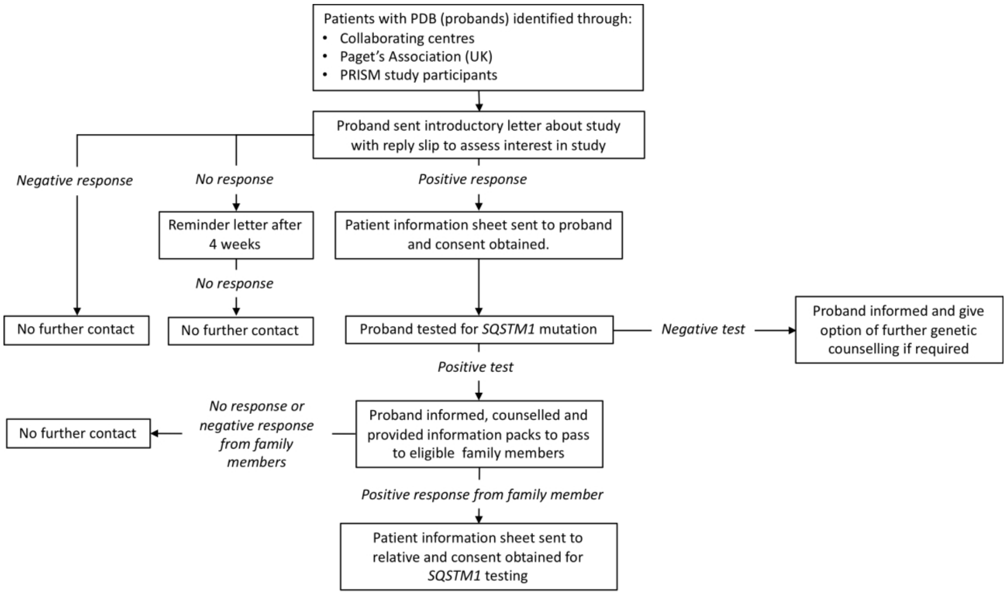

Figure 1

270x159mm (300 x 300 DPI)

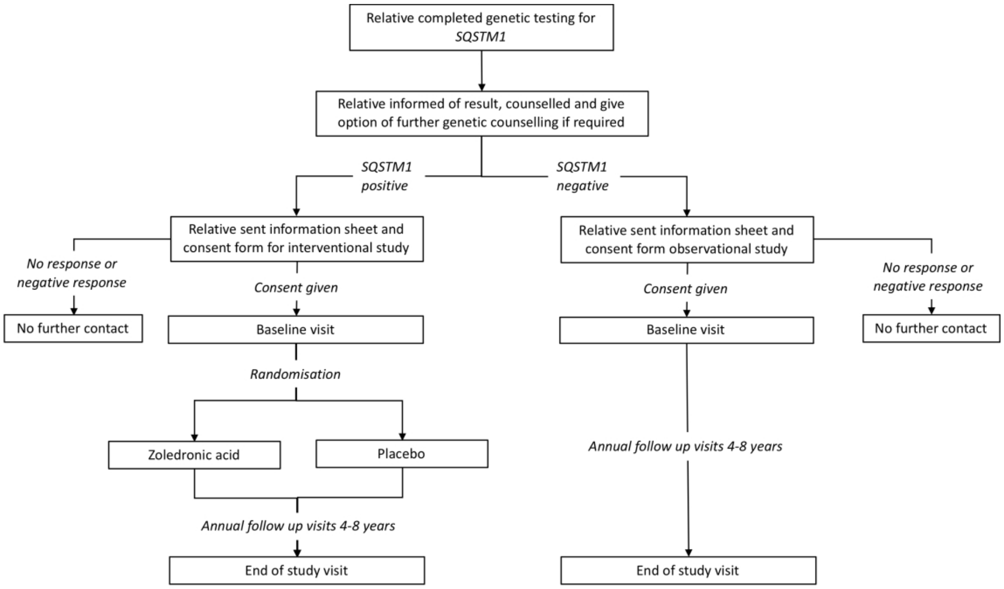

Figure 2

283x166mm (300 x 300 DPI)

STANDARD PROTOCOL ITEMS: RECOMMENDATIONS FOR INTERVENTIONAL TRIALS

SPIRIT 2013 Checklist: Recommended items to address in a clinical trial protocol and related documents*

Section/item	Item No	Description	Page
**Administrative information**			
Title	1	Descriptive title identifying the study design, population, interventions, and, if applicable, trial acronym	1
Trial registration	2a	Trial identifier and registry name. If not yet registered, name of intended registry	3
	2b	All items from the World Health Organization Trial Registration Data Set	N/A
Protocol version	3	Date and version identifier	13
Funding	4	Sources and types of financial, material, and other support	14
Roles and responsibilities	5a	Names, affiliations, and roles of protocol contributors	2, 13
	5b	Name and contact information for the trial sponsor	13
	5c	Role of study sponsor and funders, if any, in study design; collection, management, analysis, and interpretation of data; writing of the report; and the decision to submit the report for publication, including whether they will have ultimate authority over any of these activities	13
	5d	Composition, roles, and responsibilities of the coordinating centre, steering committee, endpoint adjudication committee, data management team, and other individuals or groups overseeing the trial, if applicable (see Item 21a for data monitoring committee)	11
**Introduction**			
Background and rationale	6a	Description of research question and justification for undertaking the trial, including summary of relevant studies (published and unpublished) examining benefits and harms for each intervention	5
	6b	Explanation for choice of comparators	5
Objectives	7	Specific objectives or hypotheses	6

For peer review only

Trial design	8	Description of trial design including type of trial (eg, parallel group, crossover, factorial, single group), allocation ratio, and framework (eg, superiority, equivalence, noninferiority, exploratory)	6
**Methods: Participants, interventions, and outcomes**			
Study setting	9	Description of study settings (eg, community clinic, academic hospital) and list of countries where data will be collected. Reference to where list of study sites can be obtained	7
Eligibility criteria	10	Inclusion and exclusion criteria for participants. If applicable, eligibility criteria for study centres and individuals who will perform the interventions (eg, surgeons, psychotherapists)	7
Interventions	11a	Interventions for each group with sufficient detail to allow replication, including how and when they will be administered	8,9
	11b	Criteria for discontinuing or modifying allocated interventions for a given trial participant (eg, drug dose change in response to harms, participant request, or improving/worsening disease)	N/A
	11c	Strategies to improve adherence to intervention protocols, and any procedures for monitoring adherence (eg, drug tablet return, laboratory tests)	N/A
	11d	Relevant concomitant care and interventions that are permitted or prohibited during the trial	10
Outcomes	12	Primary, secondary, and other outcomes, including the specific measurement variable (eg, systolic blood pressure), analysis metric (eg, change from baseline, final value, time to event), method of aggregation (eg, median, proportion), and time point for each outcome. Explanation of the clinical relevance of chosen efficacy and harm outcomes is strongly recommended	11
Participant timeline	13	Time schedule of enrolment, interventions (including any run-ins and washouts), assessments, and visits for participants. A schematic diagram is highly recommended (see Figure)	24, 25 (Figures 1 & 2)
Sample size	14	Estimated number of participants needed to achieve study objectives and how it was determined, including clinical and statistical assumptions supporting any sample size calculations	11, 12

For peer review only - http://bmjopen.bmj.com/site/about/guidelines.xhtml

Recruitment	15	Strategies for achieving adequate participant enrolment to reach target sample size	12

Methods: Assignment of interventions (for controlled trials)

Allocation:

Sequence generation	16a	Method of generating the allocation sequence (eg, computer-generated random numbers), and list of any factors for stratification. To reduce predictability of a random sequence, details of any planned restriction (eg, blocking) should be provided in a separate document that is unavailable to those who enrol participants or assign interventions	8
Allocation concealment mechanism	16b	Mechanism of implementing the allocation sequence (eg, central telephone; sequentially numbered, opaque, sealed envelopes), describing any steps to conceal the sequence until interventions are assigned	8
Implementation	16c	Who will generate the allocation sequence, who will enrol participants, and who will assign participants to interventions	8
Blinding (masking)	17a	Who will be blinded after assignment to interventions (eg, trial participants, care providers, outcome assessors, data analysts), and how	8
	17b	If blinded, circumstances under which unblinding is permissible, and procedure for revealing a participant's allocated intervention during the trial	9

Methods: Data collection, management, and analysis

Data collection methods	18a	Plans for assessment and collection of outcome, baseline, and other trial data, including any related processes to promote data quality (eg, duplicate measurements, training of assessors) and a description of study instruments (e.g. questionnaires, laboratory tests) along with their reliability and validity, if known. Reference to where data collection forms can be found, if not in the protocol	10,11
	18b	Plans to promote participant retention and complete follow-up, including list of any outcome data to be collected for participants who discontinue or deviate from intervention protocols	10
Data management	19	Plans for data entry, coding, security, and storage, including any related processes to promote data quality (eg, double data entry; range checks for data values). Reference to where details of data management procedures can be found, if not in the protocol	10

Statistical methods	20a	Statistical methods for analysing primary and secondary outcomes. Reference to where other details of the statistical analysis plan can be found, if not in the protocol	12,13
	20b	Methods for any additional analyses (eg, subgroup and adjusted analyses)	12,13
	20c	Definition of analysis population relating to protocol non-adherence (eg, as randomised analysis), and any statistical methods to handle missing data (eg, multiple imputation)	12,13
**Methods: Monitoring**			
Data monitoring	21a	Composition of data monitoring committee (DMC); summary of its role and reporting structure; statement of whether it is independent from the sponsor and competing interests; and reference to where further details about its charter can be found, if not in the protocol. Alternatively, an explanation of why a DMC is not needed	12
	21b	Description of any interim analyses and stopping guidelines, including who will have access to these interim results and make the final decision to terminate the trial	N/A
Harms	22	Plans for collecting, assessing, reporting, and managing solicited and spontaneously reported adverse events and other unintended effects of trial interventions or trial conduct	13
Auditing	23	Frequency and procedures for auditing trial conduct, if any, and whether the process will be independent from investigators and the sponsor	12
**Ethics and dissemination**			
Research ethics approval	24	Plans for seeking research ethics committee/institutional review board (REC/IRB) approval	15
Protocol amendments	25	Plans for communicating important protocol modifications (eg, changes to eligibility criteria, outcomes, analyses) to relevant parties (eg, investigators, REC/IRBs, trial participants, trial registries, journals, regulators)	N/A
Consent or assent	26a	Who will obtain informed consent or assent from potential trial participants or authorised surrogates, and how (see Item 32)	7
	26b	Additional consent provisions for collection and use of participant data and biological specimens in ancillary studies, if applicable	N/A

For peer review only - http://bmjopen.bmj.com/site/about/guidelines.xhtml

Confidentiality	27	How personal information about potential and enrolled participants will be collected, shared, and maintained in order to protect confidentiality before, during, and after the trial	
Declaration of interests	28	Financial and other competing interests for principal investigators for the overall trial and each study site	
Access to data	29	Statement of who will have access to the final trial dataset, and disclosure of contractual agreements that limit such access for investigators	N/A
Ancillary and post-trial care	30	Provisions, if any, for ancillary and post-trial care, and for compensation to those who suffer harm from trial participation	N/A
Dissemination policy	31a	Plans for investigators and sponsor to communicate trial results to participants, healthcare professionals, the public, and other relevant groups (eg, via publication, reporting in results databases, or other data sharing arrangements), including any publication restrictions	15
	31b	Authorship eligibility guidelines and any intended use of professional writers	15
	31c	Plans, if any, for granting public access to the full protocol, participant-level dataset, and statistical code	15
**Appendices**			
Informed consent materials	32	Model consent form and other related documentation given to participants and authorised surrogates	
Biological specimens	33	Plans for collection, laboratory evaluation, and storage of biological specimens for genetic or molecular analysis in the current trial and for future use in ancillary studies, if applicable	10,11

*It is strongly recommended that this checklist be read in conjunction with the SPIRIT 2013 Explanation & Elaboration for important clarification on the items. Amendments to the protocol should be tracked and dated. The SPIRIT checklist is copyrighted by the SPIRIT Group under the Creative Commons "Attribution-NonCommercial-NoDerivs 3.0 Unported" license.

For peer review only

For peer review only - http://bmjopen.bmj.com/site/about/guidelines.xhtml
